# Dynamic Ligand Modulation of EPO Receptor Pools, and Dysregulation by Polycythemia-Associated EPOR Alleles

**DOI:** 10.1371/journal.pone.0029064

**Published:** 2012-01-12

**Authors:** Seema Singh, Rakesh Verma, Anamika Pradeep, Karen Leu, R. Bruce Mortensen, Peter R. Young, Miho Oyasu, Peter J. Schatz, Jennifer M. Green, Don M. Wojchowski

**Affiliations:** 1 Center of Excellence in Stem Cell Biology and Regenerative Medicine, Maine Medical Center Research Institute, Scarborough, Maine, United States of America; 2 Affymax, Inc., Palo Alto, California, United States of America; Duke University, United States of America

## Abstract

Erythropoietin (EPO) and its cell surface receptor (EPOR) are essential for erythropoiesis; can modulate non-erythroid target tissues; and have been reported to affect the progression of certain cancers. Basic studies of EPOR expression and trafficking, however, have been hindered by low-level EPOR occurrence, and the limited specificity of anti-EPOR antibodies. Consequently, these aspects of EPOR biology are not well defined, nor are actions of polycythemia- associated mutated EPOR alleles. Using novel rabbit monoclonal antibodies to intracellular, PY- activated and extracellular EPOR domains, the following properties of the endogenous hEPOR in erythroid progenitors first are unambiguously defined. 1) High- Mr EPOR forms become obviously expressed only when EPO is limited. 2) EPOR-68K plus -70K species sequentially accumulate, and EPOR-70K comprises an apparent cell surface EPOR population. 3) Brefeldin A, N-glycanase and associated analyses point to EPOR-68K as a core-glycosylated intracellular EPOR pool (of modest size). 4) In contrast to recent reports, EPOR inward trafficking is shown (in UT7epo cells, and primary proerythroblasts) to be sharply ligand-dependent. Beyond this, when C-terminal truncated hEPOR-T mutant alleles as harbored by polycythemia patients are co-expressed with the wild-type EPOR in EPO-dependent erythroid progenitors, several specific events become altered. First, EPOR-T alleles are persistently activated upon EPO- challenge, yet are also subject to apparent turn-over (to low-Mr EPOR products). Furthermore, during exponential cell growth EPOR-T species become both over-represented, and hyper-activated. Interestingly, EPOR-T expression also results in an EPO dose-dependent loss of endogenous wild-type EPOR's (and, therefore, a squelching of EPOR C-terminal- mediated negative feedback effects). New knowledge concerning regulated EPOR expression and trafficking therefore is provided, together with new insight into mechanisms via which mutated EPOR-T polycythemia alleles dysregulate the erythron. Notably, specific new tools also are characterized for studies of EPOR expression, activation, action and metabolism.

## Introduction

Hematopoietic growth factors (HGF), and their cognate receptors (HGF-R's), exert prime regulation over stem, progenitor and peripheral blood cell levels [1], [2], [3]. Factors that regulate HGF-R expression, and cell surface receptor trafficking, are therefore of central importance for balanced hematopoiesis. Regulation over HGFR expression is dynamic, and can occur via diverse mechanisms. As recent examples, alternate splicing of c-KIT and IL3R-alpha can alter PI3K/AKT signaling [4], [5], while miR-155 targeting of IL13R-alpha1 receptors can divert macrophage to an M2/pro-Th(2) fate [6]. Dysregulated cell surface receptor expression also is associated with hematopoietic malignancies. To illustrate, IL7R-alpha, IL3R-alpha and c-KIT cell levels are dysregulated in adult ALL [7], AML progenitors [8], and AML1-ET09a [9]. Mutations in HGF receptors also occur that alter signal transduction capacities, and function. As one example, stop codon mutations in exon-10 of the thrombopoietin receptor enhance JAK/STAT signaling in a myeloproliferative disease context [10]. Such HGF-R mutations that give rise to C-terminal truncated receptor forms are also of broader occurrence. Alleles of GCSFR that encode such mutations, as a second example, are associated with congenital neutropenia [11], as well as hematopoietic stem cell hyper-expansion [12].

For the erythropoietin receptor (EPOR), a number of single- allele stop-codon mutations (predominantly in exon-8) have been described in association with primary familial and congenital polycythemia (PFCP) [13], [14], [15]. Such EPOR mutations often result in the loss of not only a C-terminal site for p85/p110 PI3K recruitment, but also PY motifs indicated to recruit one or more negative regulators as SOCS-1, SOCS-3 and/or SHP1 phosphatase [16], [17]. Such EPOR truncations (“EPOR-T” alleles), however, also may disrupt sites for EPOR internalization, ubiquitination, endosome trafficking and/or lysosome plus proteosomal processing. Uncertainty therefore exists concerning mechanisms of EPOR-T dysregulation. For the endogenous wild-type EPOR, basic aspects of expression and trafficking also remain contentious, with recent arguments made, for example, for ligand- independent [18] vs dependent- trafficking [19]. Studies of BTRC E3 ubiquitin ligase docking [20], cytoplasmic lysine mutations [21] and p85-alpha recruitment [19] have begun to provide insight into regulated EPOR transit, and have implicated the existence of interestingly complex mechanisms that regulate EPOR pools. In addition, studies in related IL5R and IL7R systems recently have suggested roles for endosome entry [22], [23] during HGF-R activation.

Towards better understanding EPOR trafficking properties, as well as properties of EPOR mutants harbored by PFCP patients, we presently have developed a novel panel of rabbit monoclonal antibodies to the hEPOR, and have used these new tools to study wild-type and truncated human EPOR expression, distinct molecular-weight forms, subcellular localization, activation, and trafficking in EPO- dependent human erythroid progenitor cell models. Our investigations first demonstrate dynamic, highly ligand- dependent EPOR trafficking to the extent that simple maintenance of cells in EPO essentially depletes EPOR's from the cell surface. Second, (and in contrast with certain prior studies) [18], [24], only a limited apparent intracellular EPOR pool is detected (and exists as a core-glycosylated EPOR-68K form). Third, truncated EPOR receptor forms appear to be only modestly attenuated in their EPO-induced internalization, but under physiological conditions accumulate as mature EPOR-T forms which further become persistently activated (in an EPO dose-dependent fashion). Interestingly, the expression of such truncated EPOR forms also markedly decreases endogenous wild-type EPOR levels. Overall, the present studies provide new insight into EPOR expression dynamics, as well as complex mechanisms via which mutated EPOR alleles can contribute to polycythemia. New tools also are advanced for studies of EPOR expression, activation, action mechanisms and trafficking.

## Materials and Methods

### Cell lines, and primary erythroid progenitor cells

UT7epo cells [25] were maintained in HEPES-buffered IMDM containing 10% FBS plus 3 U/mL EPO (Epoetin-alfa, OrthoBiotech, Raritan, NJ). HL-60 cells (ATCC, Manassas, Virginia) were maintained in IMDM plus 20% FBS. Media and FBS were from Invitrogen (Carlsbad, CA). In cytokine withdrawal experiments, washed cells were cultured for defined intervals in IMDM containing 0.2% BSA, 0.1 mM 2-mercaptoethanol, and 10 µg/mL holo-transferrin. Brefeldin-A (Sigma #B5936, St Louis, MO) was used at 7.5 µg/mL (0.1% DMSO, 20-hour exposure). Human bone marrow CD34^pos^-derived (pro)erythroblasts (Lonza) were expanded in StemPro34 medium (Invitrogen) supplemented with 100 ng/mL rhSCF, 20 ng/mL rhIL3, 20 ng/mL rhIL6, 100 ng/mL rhFLT3, 2.5 U/mL EPO, 0.1 µM beta estradiol and 1 µM dexamethasone. Cells were seeded at 3.5×10^6^ cells per mL. On days 1.5, 4.5, 7.5 and 10.5, one-volume of fresh medium was added. On days 3, 6, 9 and 12, 90% of medium was replaced.

### EPOR antibodies

EPOR antibodies were generated (in collaboration with Epitomics, Burlingame, CA) by immunizing rabbits with a recombinant hEPOR extracellular domain, or with KLH-coupled EPOR G334–L350 peptides [G^336^SEHAQDT-(P)-YLVLDKWL^351^]. Following a fifth boost, antigen specific immune responses were initially evaluated by ELISA (using phosphorylated and non-phosphorylated peptides). Rabbits with robust responses were used to generate hybridomas, and monoclonal antibodies. Select MAb-secretory hybridomas were expanded, and secreted IgG antibodies were purified by affinity chromatography (Protein-A-Sepharose CL4B) (Sigma).

### EPOR constructs and retroviral transduction

Using a wild-type human EPOR cDNA as a template, mutated cDNAs were prepared to correspond to those harbored by two characterized families with primary familial and congenital polycythemia [13], [14]. Each mutation is within exon-8, and each introduces a premature translational stop codon. Specific mutations are G5881T, and G1251T [13], [14]. Encoded truncated receptors (EPOR-T's) therefore lack 110 and 92 C-terminal amino acid residues respectively; include Y285 and Y344 residues in their mature forms; and are designated as EPOR-T374, and EPOR-T-392. Mutagenesis was accomplished using a Quick-Change XL system (Stratagene, Santa Clara, CA). Products were sequenced on each strand, and cDNAs were cloned to pMSCVneo (Clontech, Mountain View, CA). VSV-G packaged pMSCVneo retroviruses then were prepared as described [26], and were concentrated (12.5-fold) using a PEG method (SBI #LV810A-1, Mountain View, CA). UT7epo cells were then transduced at varied MOI's (3-fold dilution series). Specifically, exponentially growing cells were plated at 1×10^5^ cells/mL (0.5 mL, 12-well format). Polybrene (8 ug/mL) and virus then were added stepwise. At 20 hours, cells were transferred to 1 mL of fresh medium and cultured for 24 hours. During subsequent expansions, G418 selection was applied (0.9 mg/mL). An essentially equivalent approach was used to transduce HL60 cells (0.8 mg/mL G418 selection).

### Flow cytometry

For flow cytometry, cells were washed and resuspended in 4°C PBS, 0.1% BSA containing 0.3 µg/mL Fc blocker (Stem Cell Technologies #01470, Vancouver, BC). After 5 minutes of incubation (4°C), hIgG was added (50 µg/mL, 15 minutes). Cells were then incubated for 1 hour at 4°C with primary anti-EPOR antibodies (typically, 1.0 µg/0.2 mL). Washed cells were next incubated at 4°C for 30 minutes with 0.1 µg/mL Alexafluor-647 goat anti-rabbit IgG (Invitrogen #A21245, Carlsbad, CA) in PBS, 0.1% BSA. For hCD34^pos^- derived cells, transferrin receptors and glycophorin-A levels were assayed using FITC-anti-TFR, and PE-anti-GPA antibodies (BD Biosciences). Equivalent numbers of gated events were then analyzed (BD Biosciences FACScalibur flow cytometer, CellQuest Pro software) [27].

### Western blot analyses

Cell lysate preparations, SDS-PAGE, western blotting and ECL were as described previously [26], [27]. Endoglycosidase-F (New England Biolabs, Ipswich, MA) was used as prescribed (25,000 U/mL, 37°C, 3 hours). Commercial primary antibodies were from Cell Signaling Technology (Danvers, MA) and included anti- JAK2 (#3230), anti- PY1007/1008 JAK2 (#3776), and anti- beta tubulin (#2128). Signals were analyzed quantitatively using Image-J software (http://rsb.info.nih.gov/ij/). Immunoprecipitations used 0.4% Igepal cell lysates, 2×10^7^ cells per sample, and 4 µg of designated anti-EPOR antibodies. Immune complexes were retrieved using Protein-A Sepharose CL4B.

### RT-PCR

Cells were lysed directly in Trizol reagent, and RNA was isolated as previously detailed [26], [27]. Quantitative RT-PCR utilized iQ SYBR Green reagent [26], [27], a BioRad i-cycler (Model iQ5) (BioRad, Hercules, CA), and hEPOR and beta-actin primers from SuperArray (cat # PPH02642A and PPH00073E).

### Immunohistochemistry

For immunohistochemistry (IHC) cell pellets were prepared by rapidly freezing centrifuged cell pellets (dry ice). Cell pellets were then dislodged from tubes; mounted (cryomold with OCT compound) (Sakura Finetek, Torrance, CA); and immediately frozen (dry ice/isopentane). Sections (5 µm) were then cut (Leica CM1950 cryostat) (Bannockburn, IL), mounted on glass slides, and fixed (4% paraformaldehyde, 10 minutes). Endogenous peroxidases were inhibited with Peroxidazed-1 (BioCareMedical, Concord, CA), and background was blocked (Background Sniper, BioCareMedical). Tissue sections were then stained with primary antibody (5 µg/mL, 30 minutes) plus Mach2 rabbit polymer (BioCareMedical). This was followed by development with 3,3′-Diaminobenzidine (DAB, BioCareMedical) for primary antibody detection. Slides were counter-stained with hematoxylin, followed by dehydration with graded alcohol. Slides were mounted with Entellan mounting media (Electron Microscopy Sciences, Hatfield, Pa), and visualized with a Leica DM2500 microscope plus LAS software (Leica).

## Results

### EPO- exposure sharply limits the expression of high molecular weight hEPOR forms

**Figure 1:**
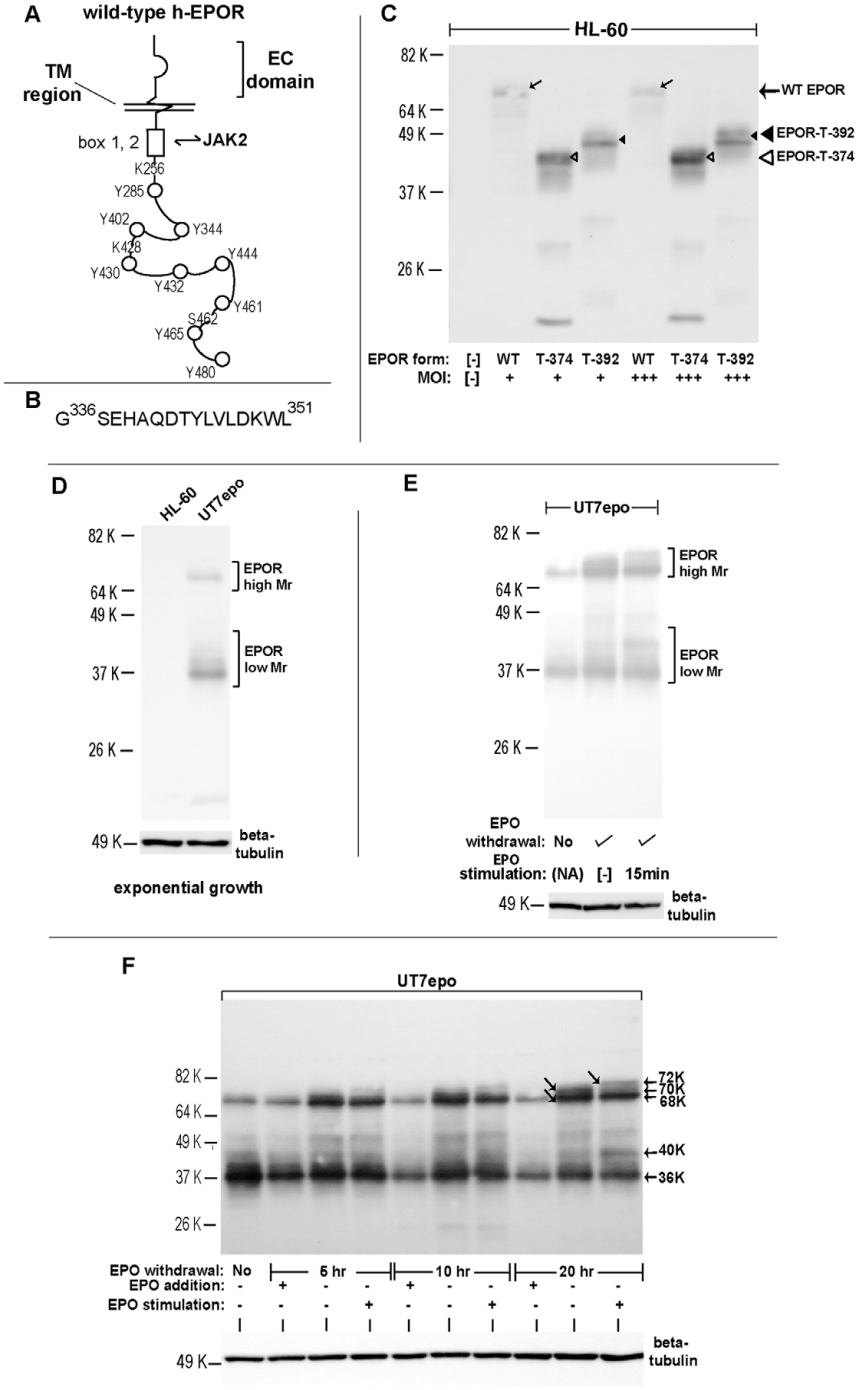
Initial defining of endogenous EPOR forms (Mr species), and expression dynamics related to EPO exposure. **A**] Schemata of the human EPOR – Features include extracellular and transmembrane regions (EC, TM); box 1,2 JAK2 association site; cytoplasmic phosphotyrosine (PY) motifs; candidate lysine (K) residues for ubiquitination; and a S462 BTRC E3 ligase interaction site. **B**] hEPOR epitope used to produce antibodies to an intracellular domain (EPOR anti-IC monoclonals). **C**] Specificity of antibody IC-c1.1 – Among antibodies generated against an EPOR peptide immunogen, several proved sensitive and specific in western blotting. For antibody IC-c1.1, specificity is illustrated using lysates from myeloid HL60 cells transduced at limiting MOI with pMSCV retroviruses encoding the wild-type EPOR, or truncated T-EPOR alleles EPOR-T374 or EPOR-T392. **D**] EPO-dependent erythroid progenitor cells in exponential growth-phase exhibit only minor levels of high-Mr EPOR's – Using antibody IC-c1.1, EPOR levels (and Mr species) in exponentially growing UT7epo cells initially were assessed. Total cell lysates prepared in parallel from human myeloid HL60 cells served as a negative control. In UT7epo cells, low-Mr EPOR species predominate, with only minor levels of high Mr EPOR species detected. **E**] EPO-withdrawal and -challenge experiments indicate the formation of higher-Mr EPOR species when EPO is limiting (and when EPOR^pos^ cells are challenged by EPO) – UT7epo cells were cultured for 24 hours in the absence of EPO, and then exposed to EPO (3 U/mL) for 0, or 15 minutes (center and right lanes). For comparison, EPO levels (and species) in exponentially growing UT7epo cells also were co-analyzed (left lane). **F**] Time-course of the formation of EPOR-70K receptors under limiting-EPO conditions, and EPO-induced conversion to activated EPOR-72K species – Exponentially growing UT7epo cells were transferred to IMDM, transferrin (10 ug/mL), 0.2% BSA, 0.1 mM 2-mercaptoethanol and cultured for the indicated intervals (time-points). For comparison, subcultures received EPO (“EPO addition”). Where indexed, cells subsequently were challenged with EPO (3 U/mL) for 15 minutes (“EPO stimulation”). Lysates then were prepared, and analyzed by western blotting.

Studies of the regulated expression, and trafficking, of the hEPOR have been hampered due to low-level expression of endogenous EPOR's [28], [29] and by the limited specificity of anti-EPOR antibodies [30]. To advance such studies, we first prepared novel rabbit monoclonal antibodies against an hEPOR membrane-proximal cytoplasmic motif (G^336^SEHAQDTYLVLDKWL^351^) (Figures 1A, 1B). Antibodies from several select clonal hybridomas (as generated from rabbits immunized with this EPOR epitope) proved to exhibit high specificity, and sensitivity. In Figure 1C, this is demonstrated via experiments in which myeloid HL60 cells were transduced at limiting MOI's with VSV glycoprotein-packaged pMSCVneo retroviruses encoding the wild-type hEPOR, or one (G5881T) or another (G1251T) naturally occurring C-terminal truncated EPOR allele. Within lysates prepared from these HL60-EPOR lines, predominant EPOR forms detected by western blotting (with designated MAb “IC-c1.1”) corresponded to the predicted full-length products of WT-EPOR, EPOR-T-392 or EPOR-T-374 alleles (i.e., ∼68 K, 48 K and 42 K). As estimated by flow cytometry and western blotting, ectopic EPOR expression levels in these HL60-EPOR lines was between 20% to 60% of endogenous EPOR levels in UT7epo cells (data not shown). EPOR antibody IC-c1.1 was not reactive with proteins from HL60 cell lysates. (For comparison, properties of additional, but not fully specific anti-EPOR monoclonal antibodies also are illustrated in Figure S1).

Next, using anti-EPOR antibody IC-c1.1, dynamics of endogenous EPOR expression were assessed in exponentially growing EPO- dependent human erythroid UT7epo cells. Somewhat unexpectedly, only limited levels of high Mr EPOR forms were detected and instead, a Mr ∼36 K EPOR species predominated (Figure 1D, right lane). In contrast, when EPO was withdrawn, substantial increases in levels of high Mr EPOR forms resulted (Figure 1E). When cells subsequently were re-exposed to EPO (3 U/mL, 15 minutes) a slower migrating higher Mr EPOR form was observed (Figure 1E, right lane). Levels of Mr ∼36 K and 40 K processed EPOR forms also increased, which are proposed to correspond to processed apparent EPOR turnover products.

These initial experiments indicated that endogenous EPOR levels in human erythroid progenitor cells might be markedly subject to ligand regulation. This concept was assessed further in experiments that examined time-component effects of limiting EPO on EPOR expression. At staggered intervals UT7epo cells were washed, replated in the absence of EPO, and cultured for 5, 10 or 20 hours prior to lysate preparation and western blotting for EPOR forms. As one control, EPO was added back (included) in cultures of washed and re-plated cells (“EPO addition”). In addition, at the end of each time interval of EPO- withdrawal, cells were challenged with EPO (3 U/mL) for 15 minutes prior to lysis (“EPO stimulation”). At 5, 10 and 20 hours of culture in the absence of EPO, major increases in levels of a Mr ∼68 K EPOR form first were observed (Figure 1F). Although challenging to resolve, at 20 hours (especially) a discrete Mr ∼70,000 EPOR species also formed in the absence of EPO (Figure 1F, 20 hr time-frame, center lane). Subsequent brief exposure to EPO (15 minutes, 3 U/mL) selectively converted this Mr ∼70 K species to an activated Mr ∼72 K EPOR form (Figure 1F, right-most lane). Therefore, the limiting of EPO leads to an accumulation of EPOR-68K and EPOR 70K forms (which otherwise comprise rare EPOR species, especially EPOR-70K).

For antibody IC-c1.1, additional data in support of specificity in western blots was provided by the ability of immunizing peptide to block the detection of the endogenous EPOR species in UT7epo cells (Figure S2). In addition, the utility of three anti-EPOR antibodies (IC-c1.1, IC-c34.11 and EC-c38.5) in immunoprecipitation assays was initially demonstrated (Figure S3) as was the application of antibody IC-c1.1 in IHC (Figure S4).

### hEPOR expression at the cell surface, and EPOR internalization, are markedly ligand-dependent

**Figure 2:**
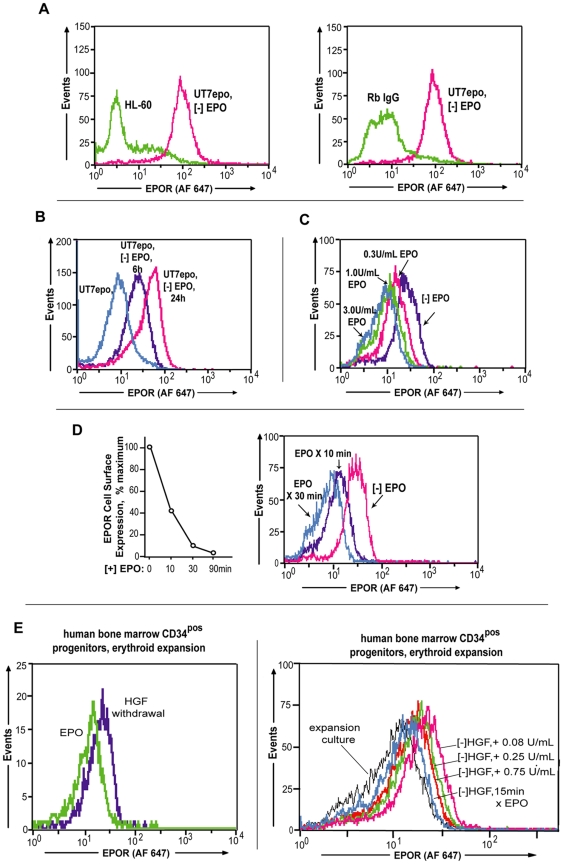
EPOR expression at the cell surface depends sharply upon the history of EPO- exposure. **A**] Cell surface EPOR detection using antibody EC-c38.5 – Among rabbit monoclonal antibodies generated against an hEPOR ECD, several proved sensitive and specific in flow cytometry assays (Alexafluor 647 second antibody detection). For EC-c38.5, this is illustrated by assays of endogenous EPOR's on UT7epo cells, here following brief EPO withdrawal (8-hours). Negative controls included myeloid HL60 cells, and non-immune rabbit IgG. **B**] Low ligand levels allow for EPOR expression at the cell surface – In UT7epo cells, cell surface EPOR levels were assayed at 6 hours or 24 hours following EPO withdrawal (and were compared to levels detected during exponential growth in EPO). **C**] Effects of exposure to EPO at varied concentrations on EPOR internalization also were assessed (at 30 minutes of exposure). **D**] EPO exposure essentially depletes cell surface EPOR's – UT7epo cells were cultured without EPO for 20 hours. Cell surface EPOR levels were then assayed through time following EPO exposure (3 U/mL). **E**] Specific detection of cell surface EPOR expression in developing human CD34-derived primary erythroid progenitors – Left panel: Primary erythroid cells were expanded from human BM CD34^pos^ cells in StemPro34 medium containing SCF (100 ng/mL), IL3 (20 ng/mL), IL6 (20 ng/mL), FLT3 (100 ng/mL), EPO (2.5 U/mL), 0.1 µM beta-estradiol and 1 µM dexamethasone. For cell populations generated at 10.5 days of expansion, and following 12 hours of HGF withdrawal, cells then were exposed (+/−) to EPO for 30 minutes; adjusted to 2°C; and assayed by flow cytometry for levels of cell surface EPOR expression (anti-EPOR antibody EC-c38.5). Myeloid HL-60 cells served as a non-specific binding control. Right panel: Exposure to EPO during HGF withdrawal at doses as low as 0.25 U/mL proved to rapidly down-modulate cell surface EPOR's (for comparison, effects of 15-minute exposure to EPO at 2 U/mL post HGF-withdrawal on EPOR levels were co-analyzed).

To enable direct analyses of EPOR expression at the cell surface, rabbit monoclonal antibodies to the EPOR extracellular domain were prepared, and assessed for utility and specificity in flow cytometry. Three such antibodies proved to be sensitive, and highly specific. In the present studies, one such EPOR MAb (designated “EC-c38.5”) was used. Figure 2A illustrates specificity first by the assay of cell surface EPOR's on UT7epo cells following EPO withdrawal as compared directly to myeloid HL60 cells (left panel); and second via staining of UT7epo cells (following EPO withdrawal) with MAb EC-c38.5 (*vs* a negative control of rabbit IgG) (right panel).

Effects of EPO withdrawal on endogenous cell surface EPOR expression in UT7epo cells were next further analyzed. At 6 hours of EPO withdrawal, moderate increases in cell surface EPOR expression were detected, but levels substantially increased further at 24 hours of EPO withdrawal (Figure 2B). EPO effects on EPOR down-modulation also were studied. At 30 minutes of EPO- exposure, a clear dose-dependent effect on cell surface EPOR down-modulation was observed (Figure 2C). In related time-course analyses, when EPO-deprived UT7epo cells were challenged with EPO at a fixed concentration of 3 U/mL, >90% of EPOR's were lost from the cell surface by 30 to 90 minutes of exposure (Figure 2D). These studies are consistent with western blot analyses (see above, Figures 1D, E, F); reinforce a case that EPOR trafficking is strongly ligand-dependent; and also at least suggest (by correlation) that EPOR-70K species corresponds to a cell surface- resident EPO receptor pool.

To further establish the property of stringent ligand- dependency for cell surface EPOR expression, endogenous EPOR expression in primary human bone marrow CD34^pos^- derived erythroid progenitors also was studied. As observed for UT7epo erythroid progenitors, withdrawal of EPO resulted in multi-fold increases in mean levels of EPOR expression (Figure 2E, left panel). When exposed to EPO at limiting concentrations (during overnight culture), cell surface EPOR levels on primary pro-erythroblasts likewise were readily diminished in a clear EPO concentration- dependent fashion (Figure 2E, right panel). To our knowledge, this is the first report that allows for analyses of endogenous EPOR trafficking in primary human bone marrow proerythroblasts. In further support of these studies, western blot (and RT-PCR) analyses were performed. These studies indicated persistent expression of the hEPOR in both early and late GPA^high^ as well as GPA^low^ proerythroblasts (Figure S5).

### Defining the nature of endogenous EPOR Mr species

**Figure 3:**
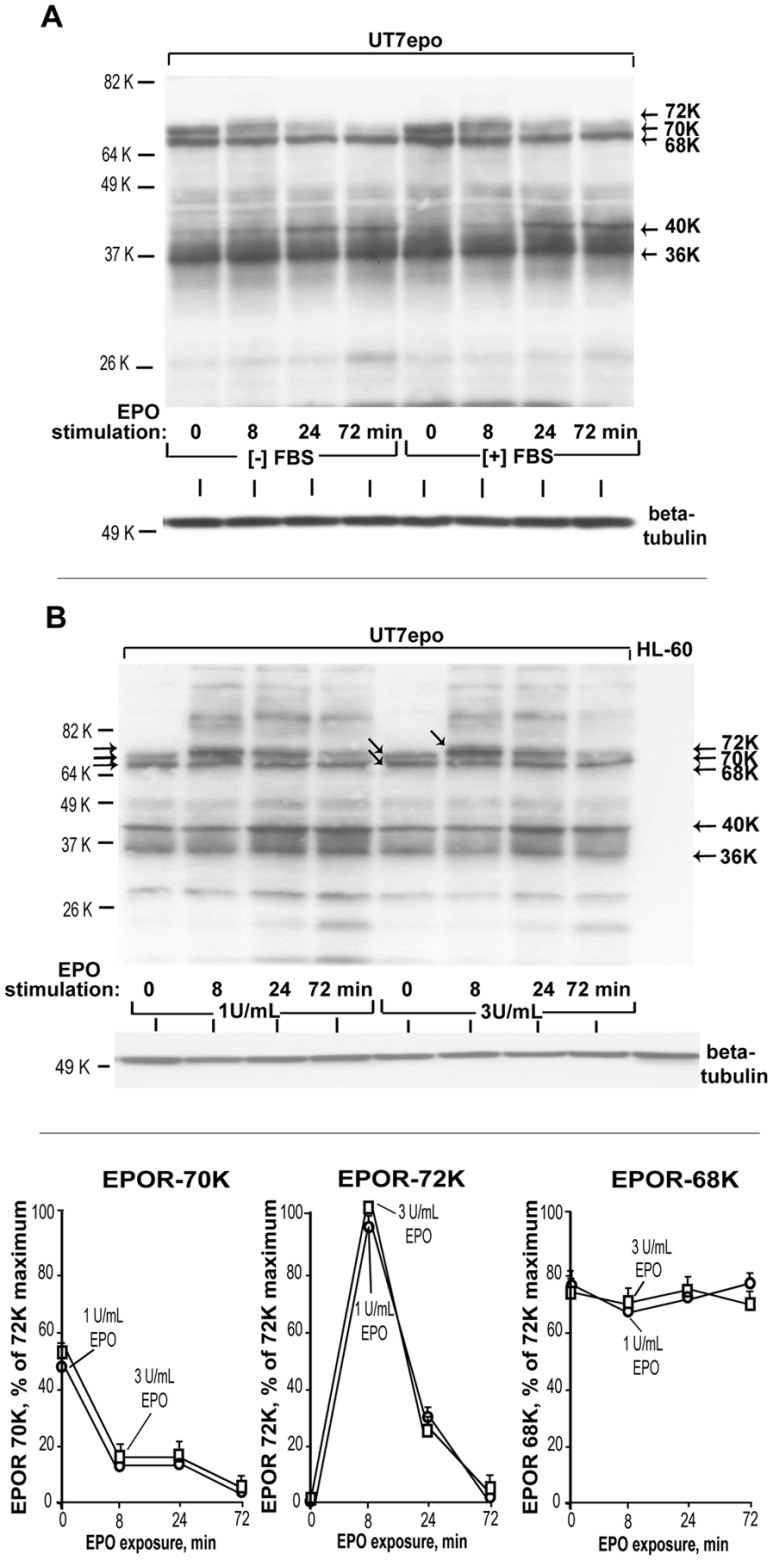
Initial defining of proposed intacellular, cell surface and ligand- activated EPOR forms. **A**] Apparent conversion of an EPOR-70K form (but not an EPOR-68K form) to an activated EPOR-72K species– UT7epo cells were cultured for 20 hours in the absence of EPO (and absence or presence of 10% FBS). Cells then were challenged with EPO (3 U/mL). At the time-points indicated, lysates were prepared, and analyzed for EPOR forms by western blotting. **B**] Time- dependent, and EPO dose- dependent formation of EPOR-70K and EPOR- 72K species- UT7epo cells were cultured for 20 hours in the absence of hematopoietic growth factors (to give rise to EPOR-68K and -70K species). Cells then were challenged with EPO at either 1 U/mL (left panels) or 3 U/mL (right panels). At 0, 8, 24 and 72 minutes of EPO exposure, lysates were prepared and analyzed by western blotting for EPOR forms. Kinetics of EPOR-70K loss, and EPOR-72K formation as induced by lower-dose EPO were similar to higher-dose EPO. Composite data for effects of EPO on EPOR-70K, EPOR-72K and EPOR-68K forms are also summarized quantitatively (lower panel). Values (mean expression levels) are normalized to maximal levels of activated EPOR-72K species observed. (Also note the apparent lack of EPO-effects on levels of EPOR-68K).

In examining effects of limited ligand availability on EPOR expression, we observed an apparent time-dependent increase first for an EPOR-68K receptor form, and at later time-points for an apparent EPOR-70K EPOR form (see above: Figure 1F, lane 1 *vs* lanes 3, 6, 9). Initial EPO- exposure experiments also at least suggested that EPOR-70K may correspond to a cell surface species, while EPOR-68K might represent an intracellular EPOR precursor pool. To better define the nature of observed major EPOR forms, extended EPO withdrawal- and exposure- experiments were performed (with gradient gels plus overnight electrophoresis employed in western blot analyses). At 20 hours post EPO- withdrawal, EPOR-68K and EPOR-70K forms each became clearly represented, together with a lower Mr EPOR-36K form (Figure 3A, lanes 1 and 5). Upon EPO exposure, EPOR-70K was selectively lost, and an EPOR-72K species rapidly formed (during an apparent conversion to activated EPOR's). Possible effects of including FBS during EPO- withdrawal (and exposure) also were tested, but EPOR expression patterns were not altered (Figure 3A, right lanes). EPO- exposure also generated a Mr 40 K EPOR-form, which together with EPOR-36K are indicated to represent EPOR turnover products. These results were reproducibly observed when either 1 U/mL or 3 U/mL EPO was used in EPO-challenge experiments (Figure 3B, upper panel) (note: 3 U/mL is required to maintain exponential growth and full viability of UT7epo cells). A quantitative summary of EPOR-70K, -72K and -68K expression as observed in these time-course and EPO-dosing experiments is also provided (with normalization to maximum levels observed for EPOR-72K) (Figure 3B, lower panel).

**Figure 4:**
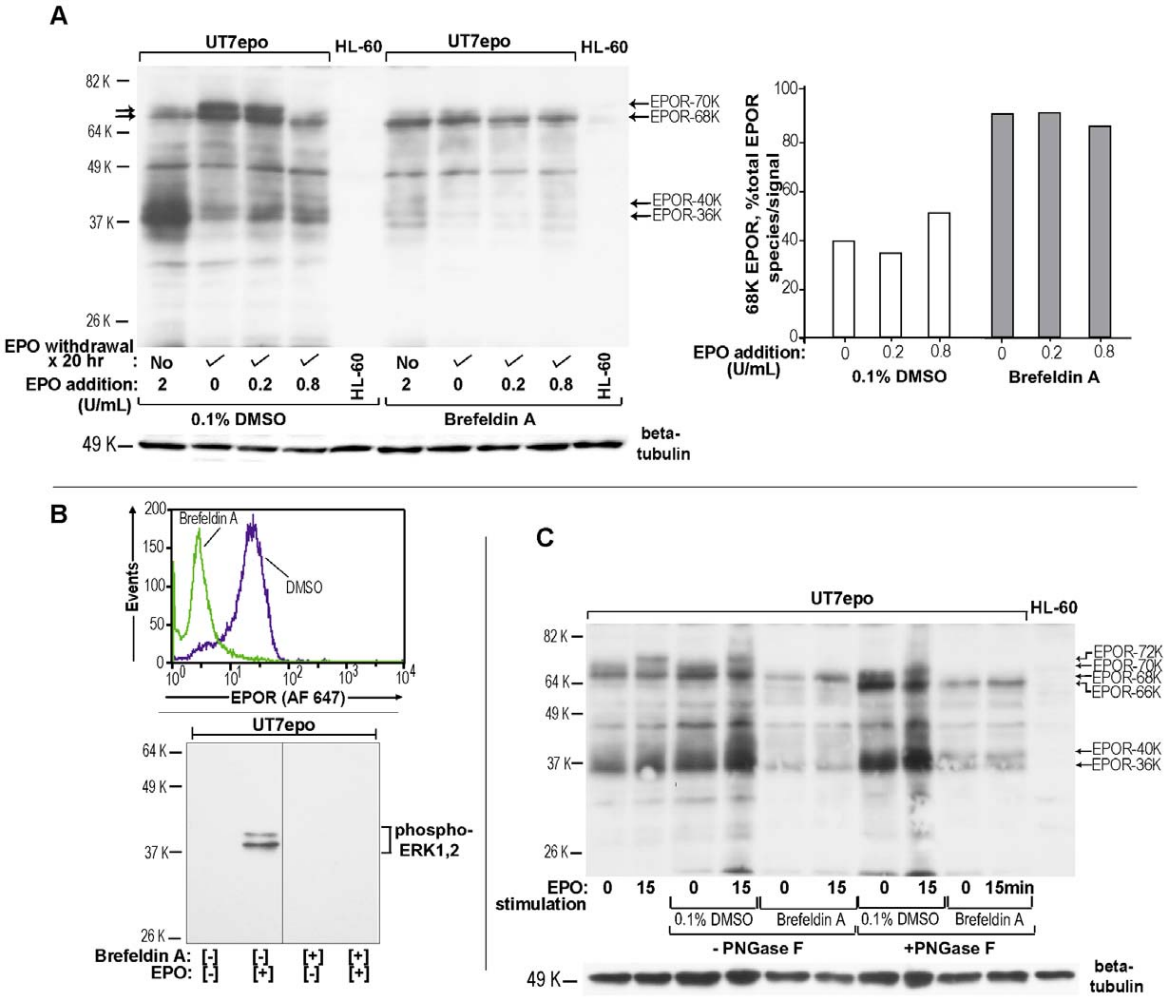
EPOR-68K species correspond to a brefeldin-resistant, endoglycosidase F- sensitive intracellular EPOR pool. **A**] Brefeldin-A effects on endogenous EPOR forms – UT7epo cells were cultured without EPO for 16 hours, and then for 20 hours in the presence or absence of brefeldin A (65 ug/mL, 0.1% DMSO) with EPO at 0, 0.2 or 0.8 U/mL. Lysates then were prepared and analyzed for EPOR forms. Exponentially growing cells (+/− brefeldin A) also were co-analyzed. In the right panel, relative levels of EPOR-68K were quantitatively estimated. **B**] Brefeldin-A inhibits cell surface EPOR expression and ERK1/2 activation– Upper panel: Following EPO-withdrawal, and [+] vs [−] brefeldin-A exposure, levels of cell surface EPOR expression were assayed. Lower panel: For cells cultured without EPO and +/− brefeldin-A, subpopulations were challenged with EPO (15 minutes). Lysates were then analyzed for levels of phosphorylated ERK1, 2. **C**] EPOR-68K is an endoglycosidase F- sensitive, apparent core glycosylated species– UT7epo cells were cultured in the absence of EPO for 20 hours +/− brefeldin A. Each treatment group was then exposed to EPO (0 or 15 minutes). Lysates were prepared, treated with endoglycosidase-F (i.e. N-glycanase) and analysed by western blotting (antibody IC-c1.1).

**Figure 5:**
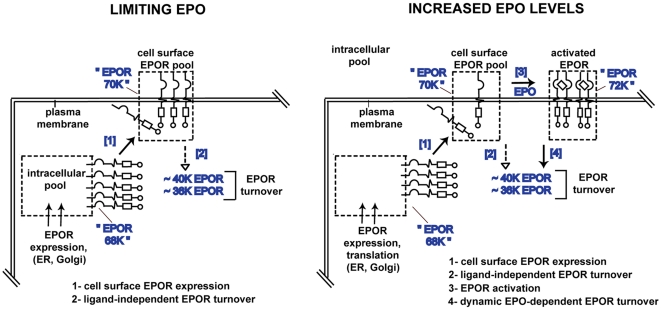
Framing a basic model for endogenous EPOR trafficking. Left panel: Data in Figures 1, 2, 3 and 4 indicate that conditions of low level EPO provide for increases in intracellular EPOR-68K and cell surface EPOR-70K pools. Right panel: This poises targeted cells for a rapid response to EPO. Ligand-induced EPOR turnover to 40 K and 36 K forms also occurs. A subpopulation of EPOR's also undergoes constitutive turnover.

In further studies of EPOR forms (and their possible inter-relatedness), effects of brefeldin-A and/or N-glycanase were analyzed (Figure 4). When UT7epo cells were exposed to brefeldin-A (an inhibitor of ER and golgi compartmentalized guanine nucleotide- exchange factors as involved in Arf1p-dependent transport) [31], an EPOR-68K form predominated. In addition, and in-keeping with the notion that EPOR-68K may comprise an intracellular EPOR pool, little to no conversion of EPOR-68K to higher Mr forms was observed upon EPO-exposure (Figure 4A). In addition, following brefeldin-A exposure and post- EPO withdrawal, UT7epo cells did not express detectable cell surface EPOR's; and did not respond to EPO with ERK1,2 activation (PT-202, PY-204 phosphorylation) (Figure 4B). Finally, lysates prepared from brefeldin-A (or DMSO-) exposed UT7epo cells were treated with N-glycanase (+/− PNGase F) prior to western blot analyses in order to assess whether EPOR-68K might represent an aglycosyl precursor. Interestingly, EPOR-68K (as well as EPOR-70K and -72K species) proved to be core-glycosylated (PNGase F sensitive), and lessened in apparent Mr by ∼2 K due to PNGase F treatment (“EPOR-66K”) (Figure 4C). Taken together, the above work allowed for the framing of models for the stringently regulated expression, and trafficking, of the endogenous hEPOR at limiting *vs* elevated EPO levels (Figure 5). Specifically, when (and only when) EPO becomes limiting, core-glycosylated apparent intracellular EPOR-68K pools first increase (Figure 5, left panel). Subsequently, EPOR-70K species are proposed to populate the cell surface, and become available for ligation. In the absence of EPO, a portion of high Mr EPOR forms nonetheless appear to be subject to ligand-independent turnover, in part, to proposed EPOR-36K and 40 Kspecies. When EPO levels increase (e.g., due to anemia or anti-anemia therapy) (Figure 5, right panel), cell surface-resident EPOR's rapidly (and transiently) convert to activated EPOR-72K species. Over a 30 to 90 minute time-course, EPOR's are then depleted from the cell surface in a ligand-dependent fashion and are processed (at least in part) to Mr 40 K and 36 K EPOR forms. (For the latter, it is noted that direct evidence has not yet been provided that these low molecular weight forms derive from ligand-activated EPOR complexes).

### Mechanisms underlying dysregulated erythroid progenitor cell expansion due to polycythemia- associated hEPOR mutations

**Figure 6:**
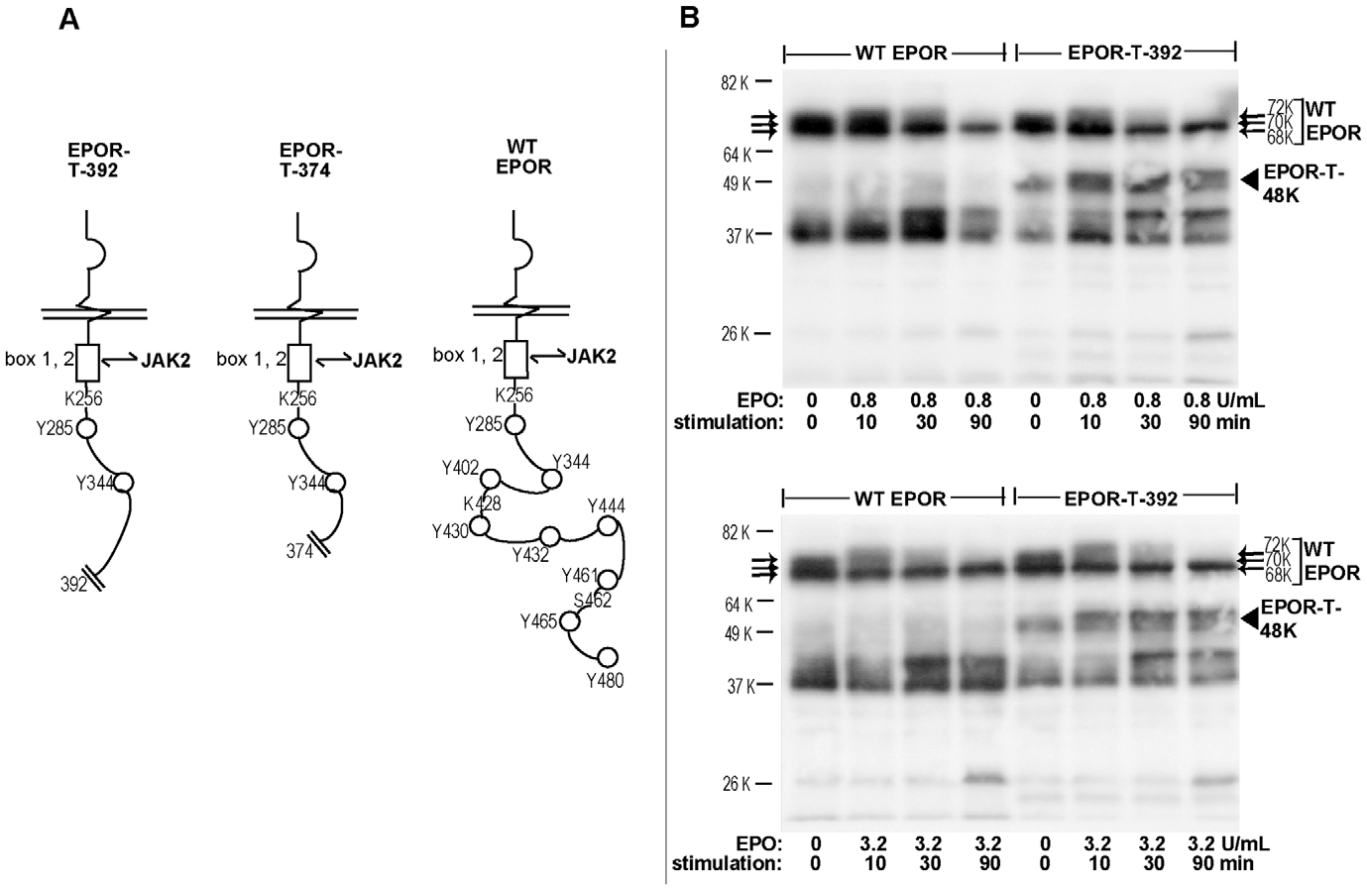
Balanced ectopic expression of the polycythemia- associated EPO receptor allele EPOR-T-392 in UT7epo cells. **A**] PFCP- associated truncated EPOR-T forms are diagrammed, together with the wild-type (wt) hEPOR. **B**] UT7epo cells were transduced at matched, low MOI's with VSVg- packaged retroviruses encoding EPOR-T-392 or, as a control for overall EPOR expression levels, the wild type EPOR (wt-EPOR). Stably transduced UT7-wtEPOR and UT7-EPOR-T-392 cells were cultured for 20 hours in the absence of EPO. Upper panel: Cells were then exposed to EPO (0.8 U/mL). At 0, 10, 30 and 90 minutes, lysates were prepared and analyzed by western blotting for levels of EPOR's. Lower panel: Analyses were as above, but with an increased EPO challenge of 3.2 U/mL.

Mutations in the EPOR that occur most frequently within exon 8 [13], [14], [15] can lead to premature stop codon utilization, and therefore co-expression of wild-type plus C-terminal truncated EPOR forms (designated “EPOR-T's”) (Figure 6A). One such recently described mutation is EPOR- G5881T [13] which encodes EPOR-T-392. Another is EPOR- G1251T [14], which encodes EPOR-T-374. To study how the truncation of such alleles might affect EPOR trafficking (and upstream signaling), pMSCVneo vectors encoding EPOR-T-392 and EPOR-T-374 EPOR alleles were used to stably transduce UT7epo cells at limiting MOI's. As an additional control (and to account for modest increases in overall EPOR levels) UT7epo cells also were transduced (in parallel at matched, limiting MOI's) with a wild-type hEPOR construct (WT-EPOR). As shown (for the wt-EPOR) in Figure 6B and Figure S6, levels of ectopically expressed EPOR's approximated those of the endogenous EPOR. Importantly, it also was possible to discern Mr species formed by EPOR-T alleles within a background of the endogenous wild-type EPOR (e.g., via densitometry scanning) (see Figure S7 for an example of EPOR-T-392). (Note: In polycythemia patients harboring truncated EPOR forms, a normal wild-type EPOR allele typically is also expressed) [13], [14], [15]. As analyzed for the polycythemia- associated EPOR allele EPOR-T-392, an additional observation was an apparent lack of any major EPO- refractive EPOR-T species (i.e., potentially corresponding to an intracellular pool).

**Figure 7:**
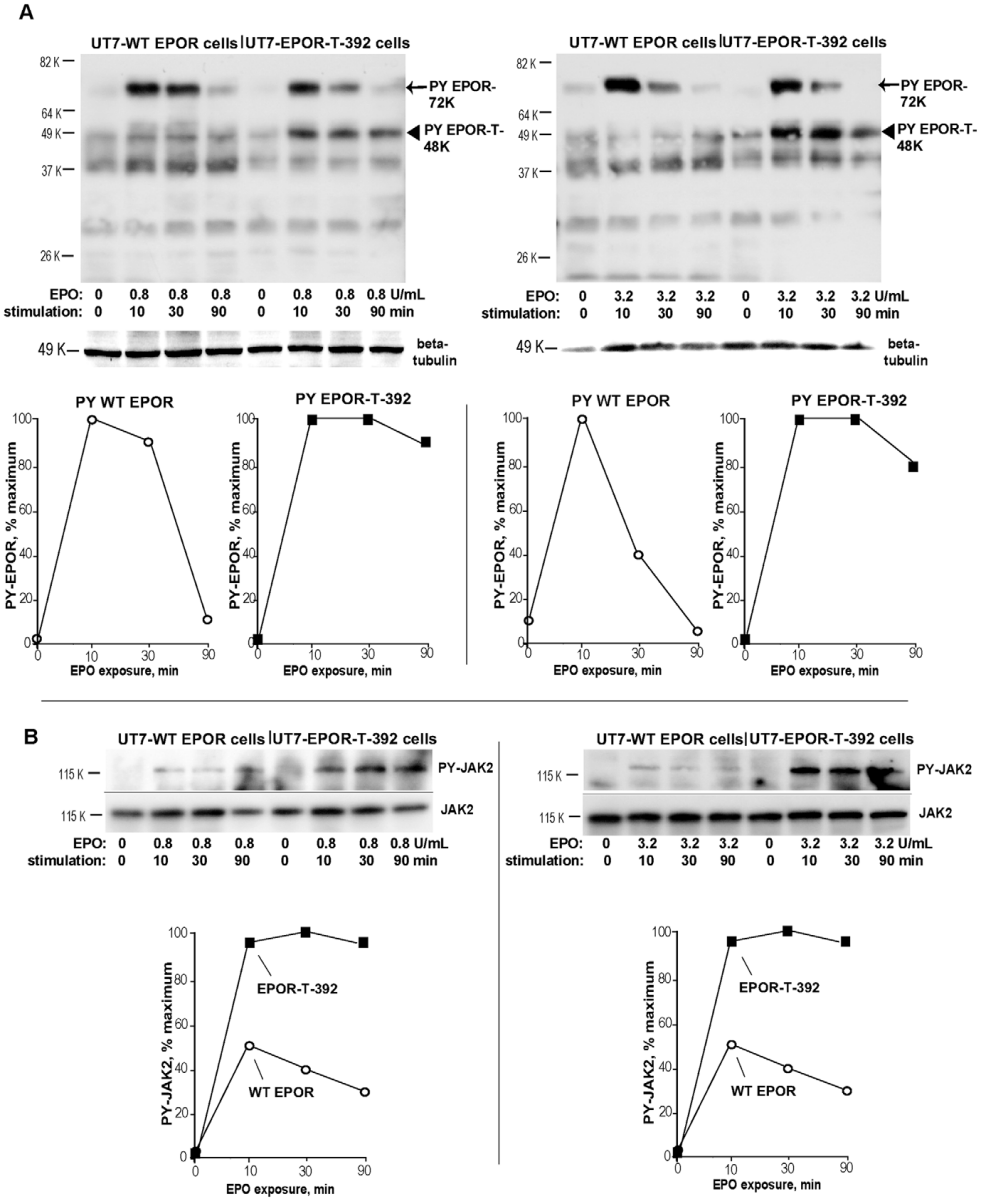
Upon EPO ligation, the polycythemia-associated truncated EPOR allele EPOR-T-392 exhibit sustained activation. **A, B**] UT7-wtEPOR and UT7-EPOR-T-392 cells were cultured for 20 hours in the absence of EPO. Cells were then exposed to EPO at 0.8 U/mL (left panel), or 3.2 U/mL (right panel). At 0, 10, 30 and 90 minutes, lysates were prepared and analyzed by western blotting for levels of PY344- EPOR (panel A). Levels of activated JAK2 also were determined (panel B). PY-EPOR and PY-JAK2 levels also were quantitatively estimated (panel B, lower sub-panels).

Using UT7-EPOR-T-392 cells, kinetics of EPOR and truncated EPOR-T allele activation next were directly assessed. This was made possible by our development of a novel rabbit monoclonal antibody to PY344- activated hEPOR's. When cells and lysates as defined in Figure 6B were analyzed (within an HGF-withdrawal plus EPO-stimulate paradigm) for levels of activated EPOR's, one obvious result was that the truncated allele EPOR-T-392 remained persistently activated for ≥90 minutes post- EPO exposure (Figure 7A, including quantitative analyses in lower panels). By direct comparison, PY-activation of the wild-type EPOR peaked within 10 minutes of EPO exposure and subsequently decayed. Further evidence for sustained activation of the truncated allele EPOR-T-392 (and its complexed co-factors) was provided by analyses of JAK2 activation (PY1007, PY1008 phosphorylation). In particular, in UT7-EPOR-T-392 cells, EPO-exposure also led to not only the sustained stimulation of JAK2, but also to increased intensities of PY-JAK2 activation (as observed at both 0.8 U/mL, and 3.2 U/mL EPO)(Figure 7B).

**Figure 8:**
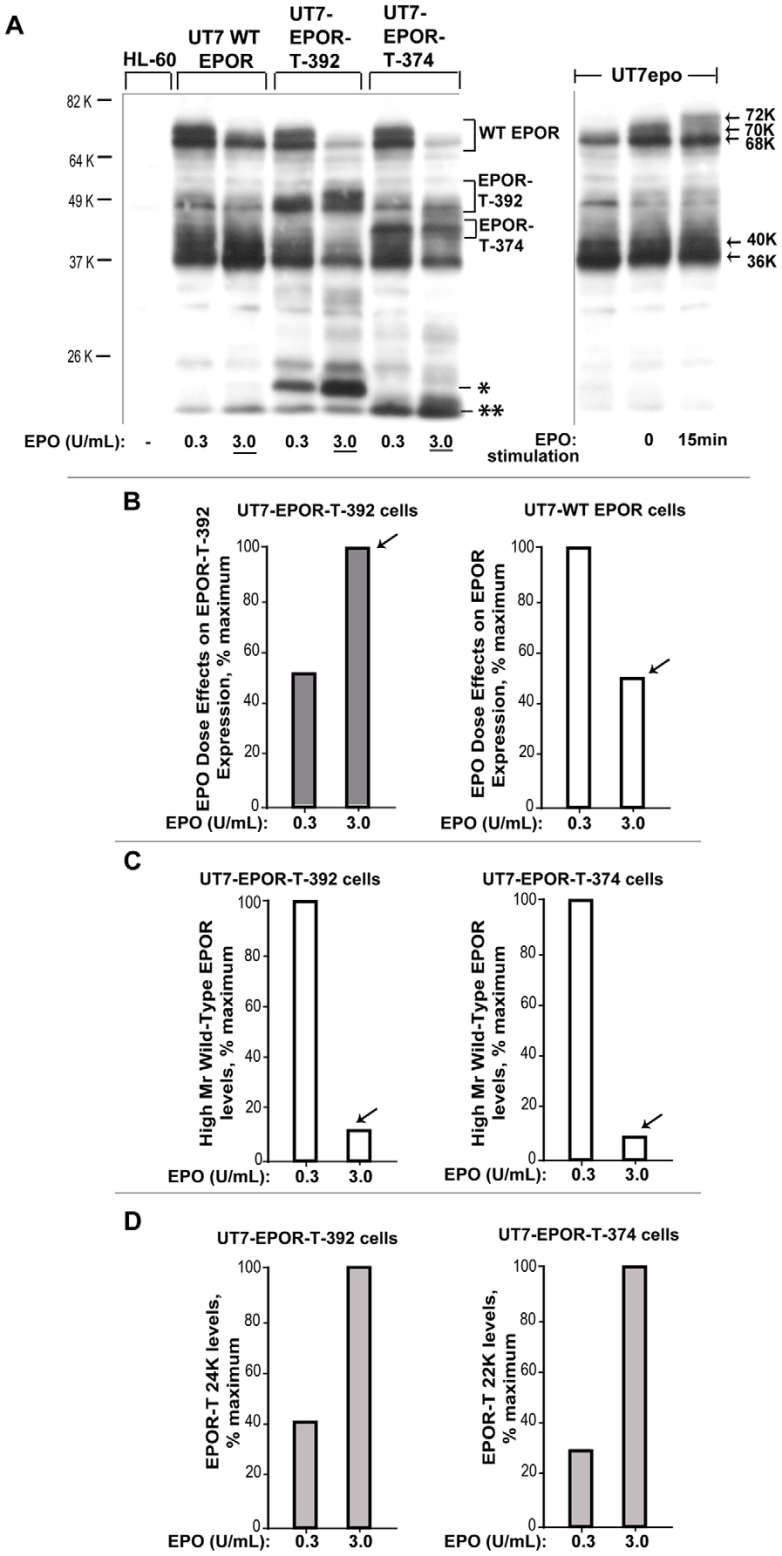
In exponentially growing erythroid progenitors, EPOR-T-392 and EPOR-T-374 accumulate, and decrease endogenous wt-EPOR expression. **A**] UT7-wtEPOR, UT7-EPOR-T392 and UT7-EPOR-T374 cells were cultured in EPO at either 0.3 or 3 U/mL. Levels of EPOR expression in directly prepared lysates then were assessed via western blotting. **B**] EPO dose-dependent increases in truncated EPOR-T-392 expression, *vs* EPO dose-dependent decreases in wild-type EPOR expression– For the above western blot analyses, quantitative imaging illustrates converse effects of EPO on expression levels of WT-EPOR *vs* EPOR-T-392 receptors. **C**] In erythroid progenitor cells expressing truncated EPOR forms, heightened EPO levels lead to marked decreases in wild-type EPOR levels– For the above western blot analyses, quantitative imaging illustrates clear inhibitory effects of EPOR-T392, and EPOR-T-374 expression on wild-type EPOR levels. **D**] Truncated alleles EPOR-T-392 and EPOR-T-374 each exhibit clear EPO dose-dependent turnover to low- Mr EPOR-T species (*,**).

A final set of experiments focused on effects of truncated EPOR-T allele expression on EPO receptor expression, and activation, under conditions of continuous UT7epo cell culture in the presence of EPO (at 0.3 or 3.0 U/mL). This approach contrasts with the above EPO withdrawal-and-stimulate design (e.g., Figure 7). Each, however, is physiologically relevant in that EPO levels in vivo can flux ≥1000-fold [18]. In these experiments, three notable results were uncovered. First, levels of high- Mr wild-type EPOR forms decreased in UT7-WT-EPOR cells due to heightened EPO dosage, while levels of truncated EPOR forms in both UT7-EPOR-T-392 and UT7-EPOR-T-374 increased (or stayed the same) with EPO dosage (Figures 8A and 8B). Second, and likewise in an EPO dose-dependent fashion, the expression of truncated EPOR forms (EPOR-T-392 or EPOR-T-374) interestingly resulted in obvious decreases in levels of the co-expressed endogenous wild-type EPOR (Figures 8A and 8C). Third (and again somewhat unexpectedly) EPO dose-dependent turnover of truncated alleles to low Mr 24 K and Mr 22 K species (respectively) was obvious (Figure 8D) (despite prior hypotheses that internalization of such truncated EPOR alleles might be disabled) [15].

**Figure 9:**
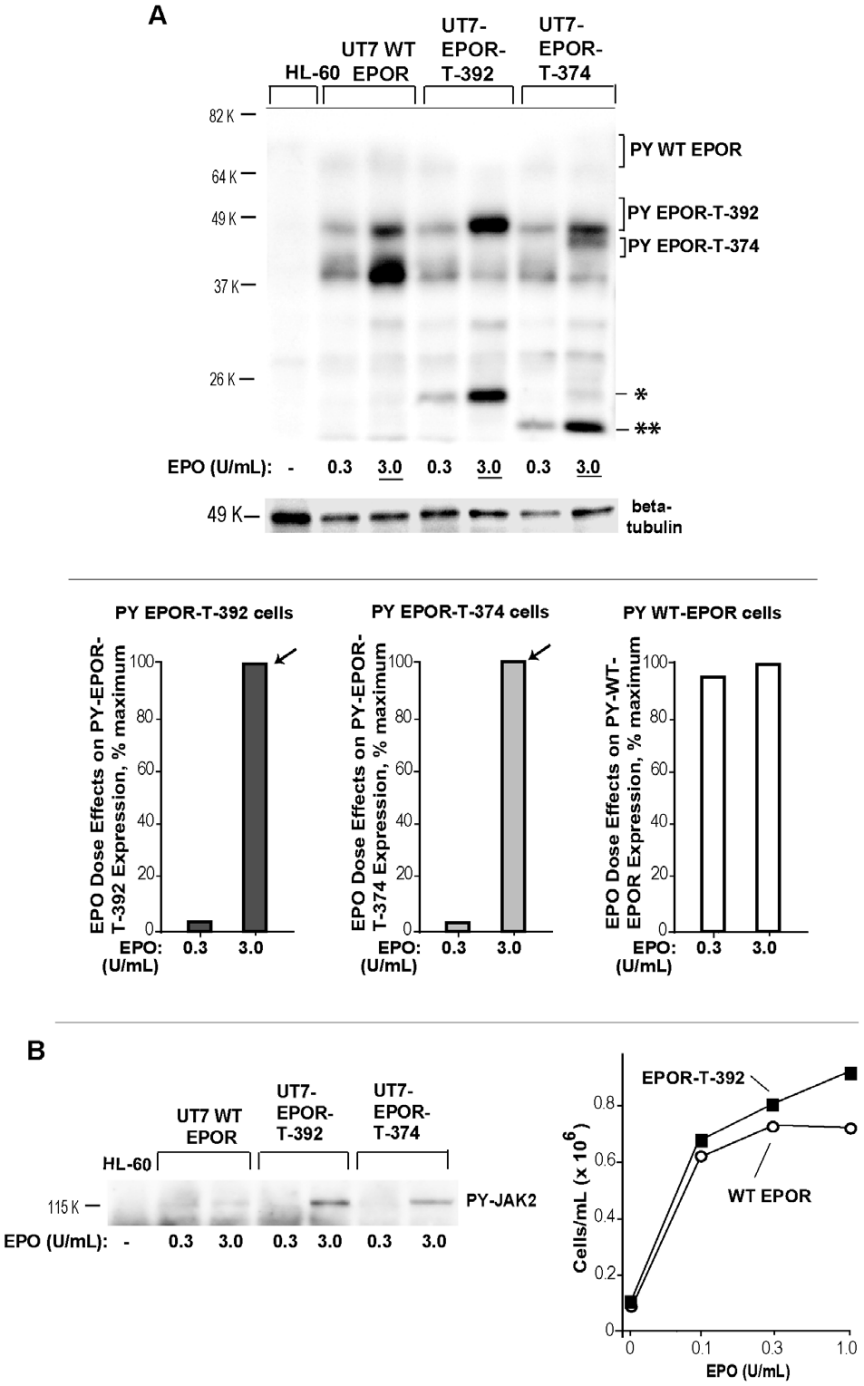
In exponentially growing erythroid progenitor cells, mature EPOR-T forms, unlike mature wild-type EPOR's, become hyper-phosphorylated. **A**] UT7-wtEPOR, UT7-EPOR-T392 and UT7-EPOR-T374 cells were cultured in EPO at either 0.3 or 3 U/mL (as detailed above, legend to Figure 8). Levels of PY-EPOR then were assessed via western blotting and also determined quantitatively (lower panel). **B**] Left panel: In the above cells (samples), levels of PY-JAK2 also were analyzed. Right panel: Heightened EPO-dependent expansion of UT7-EPOR-T392 cells. UT7epo cells expressing the wtEPOR or EPOR-T-392 at matched levels were cultured in EPO at the indicated concentrations, and at 48 hours viable cell numbers were determined.

Using our anti- EPOR-PY344 antibody, cells and lysates as studied above (Figure 8) were analyzed for levels of activated EPOR forms. Interestingly, truncated EPOR alleles (EPOR-T-392, EPOR-T-374) proved to be chronically activated (PY-344 phosphorylated) (Figures 9A), and disproportionately so as compared to overall EPOR expression patterns. Apparent proposed degradation products as Mr 24 K and 22 K species as derived from truncated EPOR-T-392, and EPOR-T-374 alleles also were obvious. For the wild-type EPOR, in contrast, only limited levels of activated high-Mr PY-EPOR forms were detected (and Mr ∼38 K and 45 K species predominated) (Figures 9A). In UT7-WT-EPOR, UT7-EPOR-T-392 and UT7-EPOR-T-374 cells, JAK2 activation also was assessed. In cells expressing C-terminal truncated EPOR alleles, JAK2 PY-phosphorylation (at PY1007, 1008) proved to be chronically elevated (Figure 9B). For EPOR-T expressing UT7epo cells, advantages in EPO-dependent expansion also were observed (Figure 9B, right panel and data not shown).

**Figure 10:**
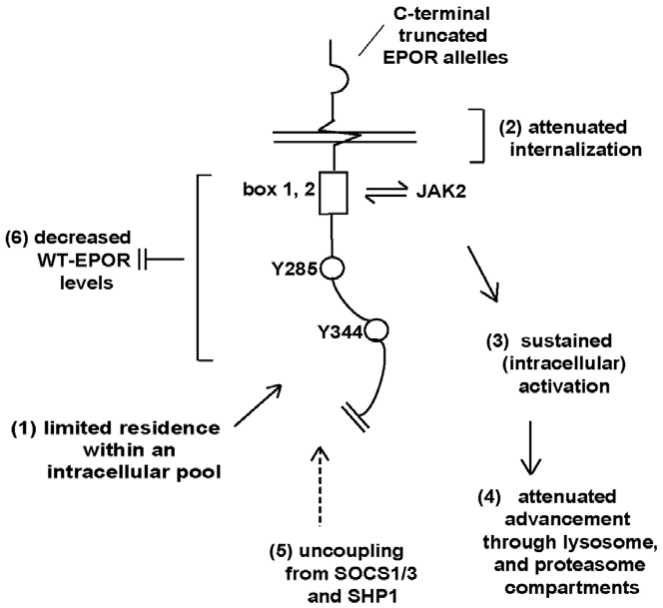
Proposed model for mechanisms that contribute to gain-of-function phenotypes exhibited by truncated PFCP EPOR alleles. For truncated EPOR alleles harbored by polycythemia patients (as diagramed) a combined set of mechanisms are proposed to lead to dysregulation of EPO/EPOR signaling (by truncated alleles, and the co-expressed wild-type EPOR). 1- Unlike the wild-type EPOR, EPOR-T alleles do not appear to accumulate within intercellular pools. 2- EPOR-T internalization is indicate to be modestly to moderately attenuated. 3- Activation of EPOR-T alleles is clearly heightened. 4- Attenuated inward trafficking also may include prolonged residence within an early lysosomal compartment. 5- As proposed previously, the truncation of C-terminal domains likely results in uncoupling from negative feed-back effects of SOCS1/3 and/or SHP1. 6- Via as yet unresolved mechanisms, expression (and ligation) of truncated EPOR alleles also results in decreased expression levels of the wild-type EPOR (as co-expressed among polycythemia patients with EPOR-T alleles).

Based on overall findings, a model is outlined in which multiple mechanisms are suggested to contribute to gain-of-function properties of polycythemia-associated C-terminally truncated, EPOR mutant alleles (Figure 10). First, unlike the wild-type EPOR, EPOR-T alleles do not appear to accumulate (or occur) as an obvious intracellular pool (component mechanism 1). Second, based on persistent expression and chronic activation as mature, activated forms, EPOR-T alleles are proposed to be attenuated in their internalization and/or trafficking through lysosomes (component mechanisms 2, 3, 4). As implicated via previous studies [16], [17], [32], [33], [34], [35], EPOR-T alleles also are less susceptible to feedback inhibition by SOCS1,3 and/or SHP1 phosphatase (component mechanism 5). In addition, co-expression of EPOR-T forms together with the endogenous wild-type EPOR (as in polycythemia patients) interestingly leads to a substantial decrease in levels of full-length EPOR's (component mechanism 6).

## Discussion

Central biological properties of hematopoietic growth factor receptors include regulated expression, cell surface residency and internalization features. These can be diverse, and are important to understand within contexts of defining cytokine target tissues and cells; possible effects of cytokine dosing on receptor up- and/or down- modulation; distinct routes engaged by growth factor agonists, mimetics or antagonists; and possible dysregulation of such receptor properties due to mutation or skewed expression. Among HGFR's, EPOR complexes present a clinically relevant system that continues to serve as an important paradigm. To illustrate, the EPOR was first to be directly associated with Janus kinase action [36]; the first HGF-R to be understood to transduce trans-membrane conformational signaling events [37]; and among the first to be defined as a target of SOCS plus SHIP negative regulators [38], [39]. Recently, new impetus to further advance an understanding of EPOR properties has been brought to bear. This includes the emergence of new EPOR agonists [40] as well as apparent EPOR- cytoprotective effects on vascular [41], retinal [42] and pancreatic beta- cells [43]. This is being met with continued new discoveries, including reports on Lnk protein [44] and transferrin receptor-2 [45] as novel transducers of EPOR action in primary proerythroblasts. With a goal of better understanding basic EPOR properties, the present study focuses first on defining the nature of distinct EPOR forms, their interrelatedness, and an unresolved question of the extent to which EPOR expression at the cell surface is subject to ligand independent- *vs* dependent- regulation.

By developing a novel panel of rabbit monoclonal antibodies specific to the hEPOR, and through analyses of the endogenous EPOR expression in an EPO- dependent human erythroid progenitor cell model, we first show that an EPOR-68K form corresponds to a core- glycosylated apparent intracellular EPOR pool; and that this pool increases in levels when EPO is limiting. Upon further sustained limiting of EPO, an EPOR-70K form is generated, which (unlike EPOR-68K) is indicated to then rapidly convert upon EPO exposure to an activated EPOR-72K species. (Interestingly, quantitative RT-PCR analyses of *EPOR* transcript levels rule out up-modulation at this level as a potential underlying mechanism) (Figure S8). Here, novel information generated is several-fold. The finding that expression levels of intracellular and apparent cell surface EPOR forms flux in ligand-dependent fashions first is insightful. Specifically, this is unlike the majority of growth factor receptors which typically maintain at least moderate pools of mature cell surface receptor forms during ligand- exposure. In studies of cells and tissues that have been exposed to EPO, lack of insight into these EPOR properties can unwittingly skew interpretations (for example, regarding the presence *vs* absence of EPOR's among candidate target cells). For apparent intracellular species, interesting questions also are raised as to what specific post-translational features may distinguish EPOR-68K from EPOR-70K forms (and which might possibly affect cell surface translocation). In addition, certain prior studies of the EPOR in Friend virus transformed MEL cells [46], or non-erythroid cells ectopically EPOR forms [18] have implicated the occurrence of large pools of intracellular EPOR's. This may relate to viral gp55 plus EPOR interactions and/or consequences of EPOR overexpression – High-level intracellular EPOR pools presently have not been observed in UT7epo erythroid progenitor cells (under any conditions).

A second major aspect of the present studies addresses the extent to which inward trafficking of EPOR's might be ligand -independent *vs* -dependent. In particular, recent studies by Becker and co-workers [18] have suggested that ligand-independent EPOR trafficking may be a prime property of the EPOR system, and that this property may allow for linear integration of EPOR signals over a broad range of ligand concentrations. In contrast, the present studies of endogenous EPOR internalization in EPO- dependent UT7epo human erythroid progenitor cells strongly argue for sharply ligand- dependent EPOR expression (and turnover) routes. This finding is based on both flow cytometry, and western blot analyses of mature EPOR forms. Furthermore, this is to the extent that cells when cultured in EPO express only nominal levels of cell surface EPOR's. Conversely, when EPO becomes limiting, endogenous EPOR forms become substantially up-modulated at the cell surface. This basic aspect of EPOR trafficking has been a matter of controversy, and is non-trivial for considerations of ligand pharmacokinetics, and responses of EPOR's and target cells to physiological 1000-fold fluxes in EPO levels [18]. Factors that may contribute to apparently disparate results and/or interpretations for ligand-modulated EPOR trafficking merit brief discussion. One may involve effects of EPOR epitope- tagging as frequently employed to overcome limiting features of available EPOR antibodies. Our laboratory was among the first to employ this approach for the EPOR in the form of an N-terminal HAI tag [47], and recently we have alternatively incorporated a FLAG tag at the hEPOR's C-terminus. Unfortunately, each modification compromises EPOR biological activity (i.e., EPO-dependenrt cell growth) several- fold as assessed in BaF3, FDCW2 and/or UT7epo cells (data not shown). This complication provided impetus for our development of new EPOR antibodies with improved properties. A second may relate to possibly altered trafficking if/when EPOR's are ectopically over-expressed. Third, analyses of EPOR trafficking in non-erythroid or non-hematopoietic cells also may be affected in unpredictable ways by heterologous factors.

The present studies also consider cell surface expression, activation and internalization properties of representative C-terminal truncated EPOR forms that have been described among PFCP patients [48]. Each form presently studied harbors a translational stop mutation within exon-8. One, G5881T, gives rise to an EPOR-T392 form [13]; and the other, G1251T, to an EPOR-T374 form [14]. In addition, each lacks 7 of 9 cytoplasmic phosphotyrosine motifs for signal transduction factor recruitment [13], [35] as well as the majority of lysine sites for potential ubiquitination. Also lacking is a proposed cytoplasmic binding motif for a BTRC E3 ubiquitin ligase [20]. One hypothesis advanced for enhanced functional attributes of EPOR-T alleles has involved a prediction that such truncated receptor forms might be substantially compromised in internalization capacities (and consequently may reside persistently on the surface of erythroid progenitors) [13], [14], [15], [48], [49]. Recent studies of mutated EPOR alleles in transfected gamma-2A and BaF3 cells also are consistent with this notion [18], [19]. As presently studied in EPO-dependent erythroid progenitors, however, internalization rates for truncated EPOR-T forms were attenuated by only ∼25% (see supporting data, Figure S9). In exponentially growing erythroid progenitors, it nonetheless was apparent that truncated EPOR-T392 and EPOR-T374 forms accumulate as mature species at levels greater than observed for the wt-EPOR. One interpretation of these results is that hyperallelic activities of truncated EPOR forms may depend on attenuated internalization (by speculation), together with possibly attenuated transit through endosomes and/or proteosomes. In addition, these defects are exerted most strikingly under physiological conditions of exponential growth. By analogy to recent findings in the JAK kinase- linked IL7R and IL5R systems, HGF receptor activation may involve (or depend upon) early endosome entry [22], [23]. If this proves to be the case for the EPOR, then attenuated EPOR-T endosomal transit might also contribute to sustained activation. Consistent with this interpretation is an observed sustained activation of PY-EPOR-T forms (together with JAK2).

An additional observation that merits discussion concerns effects of truncated EPOR alleles on the expression of the endogenous EPO receptor. (Notably, truncated EPOR alleles typically are co-expressed together with a normal EPOR allele in primary familial and congenital polycythemia patients) [14], [15], [48]. In particular, when cells were maintained in EPO, EPOR-T expression resulted in obvious decreases in endogenous EPOR levels. Several mechanisms might mediate this effect. First, when heterodimerized with truncated EPOR forms, the endogenous EPOR may be co-internalized but more readily processed (via C-terminal sub-domains) upon entering endosomes. Second, during outward trafficking, JAK2 chaperone effects, and/or EPOR processing may be affected. In support of this notion, EPOR-T effects on endogenous EPOR levels appear to also include decreases in EPOR-68K forms. Via either (or both) route(s), decreases in levels of wild-type EPOR's in erythroid progenitors of polycythemia patients translate to an increased escape of EPOR-JAK2 complexes from negative-feedback factors (as recruited via EPOR C-terminal domains). For other HGFR's that occur as truncated alleles (e.g., MPL, GCSFR) [10], [11], [12] it should be of significant interest to discover possible parallels in dysregulation in likewise clinically meaningful contexts.

A final notable advancement of the present study involves properties and utilities of our novel anti-EPOR antibodies per se. Antibody IC-c1.1 exhibits high specificity (and sensitivity) in western blotting; and antibody EC-c38.5 is proposed to possess uniquely advantageous properties for flow cytometry. Specific immunoprecipitation of EPOR's by antibodies IC-c1.1, IC-c34.11 and EC-c38.5 also is illustrated. In addition, promising utility of ICD antibody IC-c1.1 in IHC is demonstrated. Together, our new EPOR antibody panel therefore represents a valuable new tool set for investigations of the intriguing and clinically important properties of the EPOR.

## Supporting Information

Figure S1 **Apparent specificities of additional anti-EPOR ICD antibodies (and of the commercial antibody M-20).** Western blots were prepared using the cell line lysate samples indexed. Blots were then probed with the alternate anti-EPOR antibodies indicated. For comparison, data in the lower panel are provided for commercial antibody M-20.
(TIF)

Figure S2 **Detection of EPOR molecular species by anti-EPOR antibody IC-c1.1 are uniformly blocked by co-incubation with immunizing peptide.** A single gel and western blot was loaded in duplicate (left and right aspects) with the indicated HL60 cell and UT7epo cell lysates. For the half-blot in the lower panel, primary antibody IC-c1.1 was incubated with immunizing peptide (1 µM) during exposure to blotted proteins. Blots were co-processed in parallel. Loading controls are beta-tubulin (for each half blot).
(TIF)

Figure S3 **Specific immunoprecipitation of the endogenous hEPOR from UT7epo cells by anti-EPOR antibodies IC-c1.1, IC-c34.11 and EC-c38.5.** UT7epo cells were deprived of hematopoietic cytokines for 20 hours. Lysates were then prepared (Igepal, 0.4%) cleared, and incubated for 4 hours with 4 µg of anti-EPOR antibodies (or rabbit IgG). Immune complexes then were retrieved (Protein-A Sepharose CL4B), washed, eluted and analyzed by western blotting with anti-EPOR antibody IC-c1.1.
(TIF)

Figure S4 **Specific immunohistochemical assays of the human EPOR.** **A**] To allow for optimization of IHC staining (and related IHC preparations) the wild-type EPOR was ectopically expressed at limited levels in EPOR negative myeloid HL60 cells (i.e., levels estimated by western blotting to be approximately 5-fold below those of the endogenous EPOR in UT7epo cells). IHC sections then were prepared as detailed in Methods, and sections were stained with an HRP- coupled anti- rabbit IgG second antibody. **B**] Using anti-EPOR antibody IC-c1.1, specific IHC assay of the endogenous EPOR in UT7epo cells also was accomplished.
(TIF)

Figure S5 **Western blot (and parallel RT-PCR) assays of the EPOR expression in human bone marrow CD34^pos^ progenitor- derived (pro)erythroblasts.** Erythroid cells were expanded from human bone marrow CD34^pos^ progenitors in SP34ex medium. At day 10 of expansion, TFR1^high^ (TFR, transferrin receptor) cells were highly represented, and (via MACS) were enriched for GPA^low^ and GPA^high^ sub-populations (lower panel C). EPOR expression levels in each erythroid progenitor pool then were assayed by western blotting (panel A) and quantitative RT-PCR (panel B).
(TIF)

Figure S6 **Ectopic expression of EPOR's in UT7epo cells at levels approximating endogenous EPOR levels.** UT7epo cells were transduced with VSVG-packaged pMSCVneo viruses encoding hEPOR constructs or no cDNA inset (empty vector). MOI's were varied in order to determine transduction conditions that provided for the expression of EPOR alleles at physiological levels. Western blotting was with anti-EPOR antibody IC-c1.1. Data shown are for parental UT7epo cells vs. stably transduced UT7epo-wtEPOR cells. (Also see Figures 6 and 7).
(TIF)

Figure S7 **Relative mobilities (in SDS PAGE) of specific EPOR molecular weight species observed upon the expression of the wild-type EPOR and EPOR-T-392 in UT7epo cells.** For corresponding western blots, please see Figure 6B.
(TIF)

Figure S8 **EPO withdrawal does not lead to increases in EPOR transcript levels.** Exponentially growing UT7epo cells were plated at 8×10^5^ cells/mL in the absence of hematopoietic cytokines. At 0, 6, 12 and 24 hours of culture, RNA was directly isolated, EPOR transcript levels then were determined by quantitative RT-PCR. Graphed values are cycle numbers normalized for beta-actin (which varied less than 2 cycles overall among samples).
(TIF)

Figure S9 **Truncated EPOR alleles harbored by polycythemia patients are moderately attenuated in ligand-induced internalization.** **A**] Attenuated internalization of EPOR-T-392 and EPOR-T-374 alleles – UT7epo cells were transduced (at limiting MOI's) with a pMSCVneo vector encoding the wt-EPOR, EPOR-T-392 or EPOR-T-374. For cells expressing each EPOR form at low, matched levels, ligand-induced rates of EPOR internalization were determined by flow cytometry. This involved EPO withdrawal (20 hours) plus subsequent EPO challenge at either 1 U/mL or 3 U/mL. At 0, 10, 30 and 90 minutes of EPO exposure, levels of cell surface EPOR's were assayed. Truncated EPOR forms underwent internalization, but with apparently attenuated kinetics. **B**] Primary flow cytometry data are also illustrated (90 minute time points).
(TIF)
