# Supplementary figures and images for: Dynamic Ligand Modulation of EPO Receptor Pools, and Dysregulation by Polycythemia-Associated EPOR Alleles
Source: PLoS One. 2012 Jan 12;7(1):e29064. doi: 10.1371/journal.pone.0029064 (PMC3257245; doi:10.1371/journal.pone.0029064)

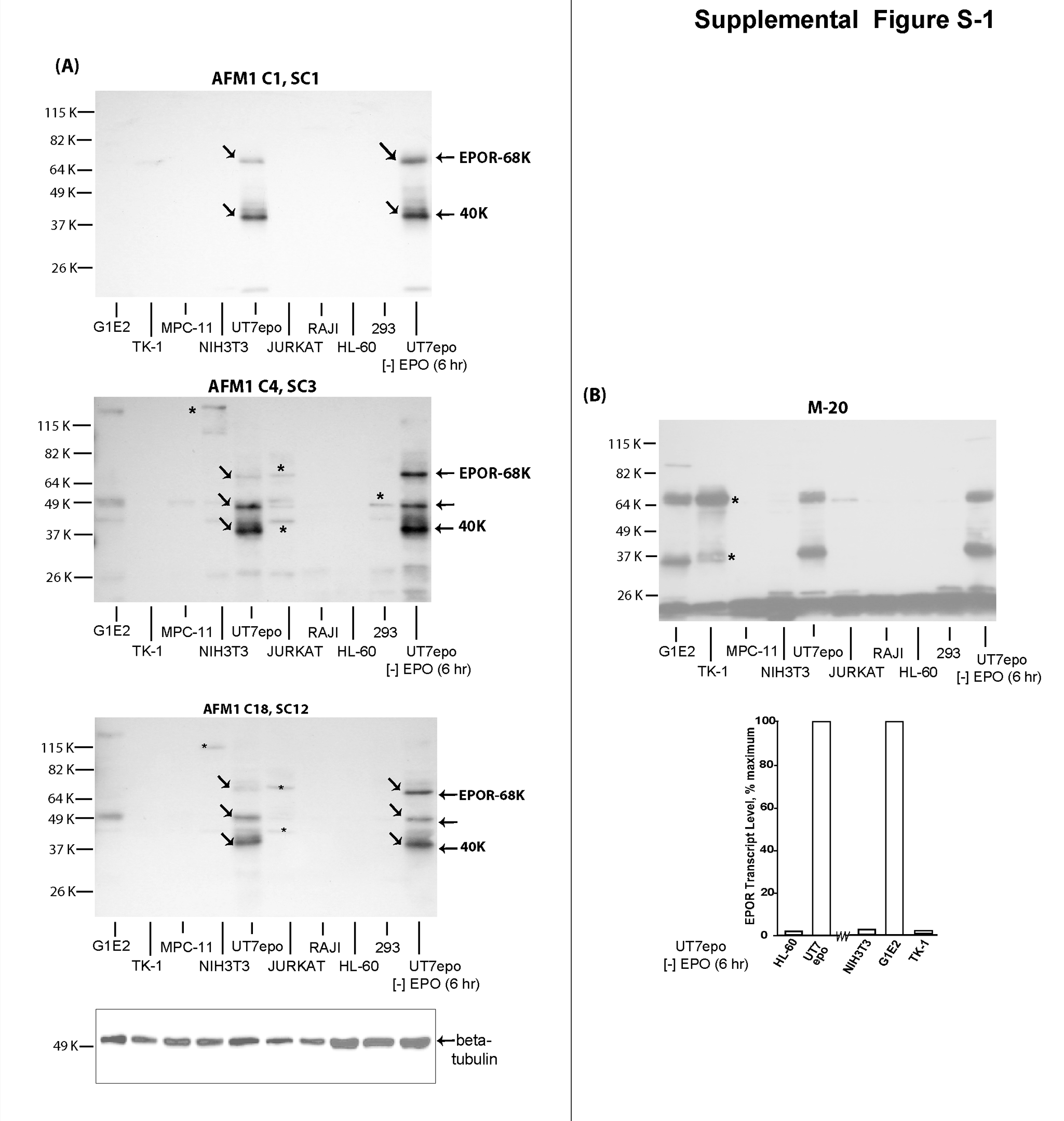

Supplement: Figure S1 — Apparent specificities of additional anti-EPOR ICD antibodies (and of the commercial antibody M-20). Western blots were prepared using the cell line lysate samples indexed. Blots were then probed with the alternate anti-EPOR antibodies indicated. For comparison, data in the lower panel are provided for commercial antibody M-20. (TIF) [file pone.0029064.s001.tif]

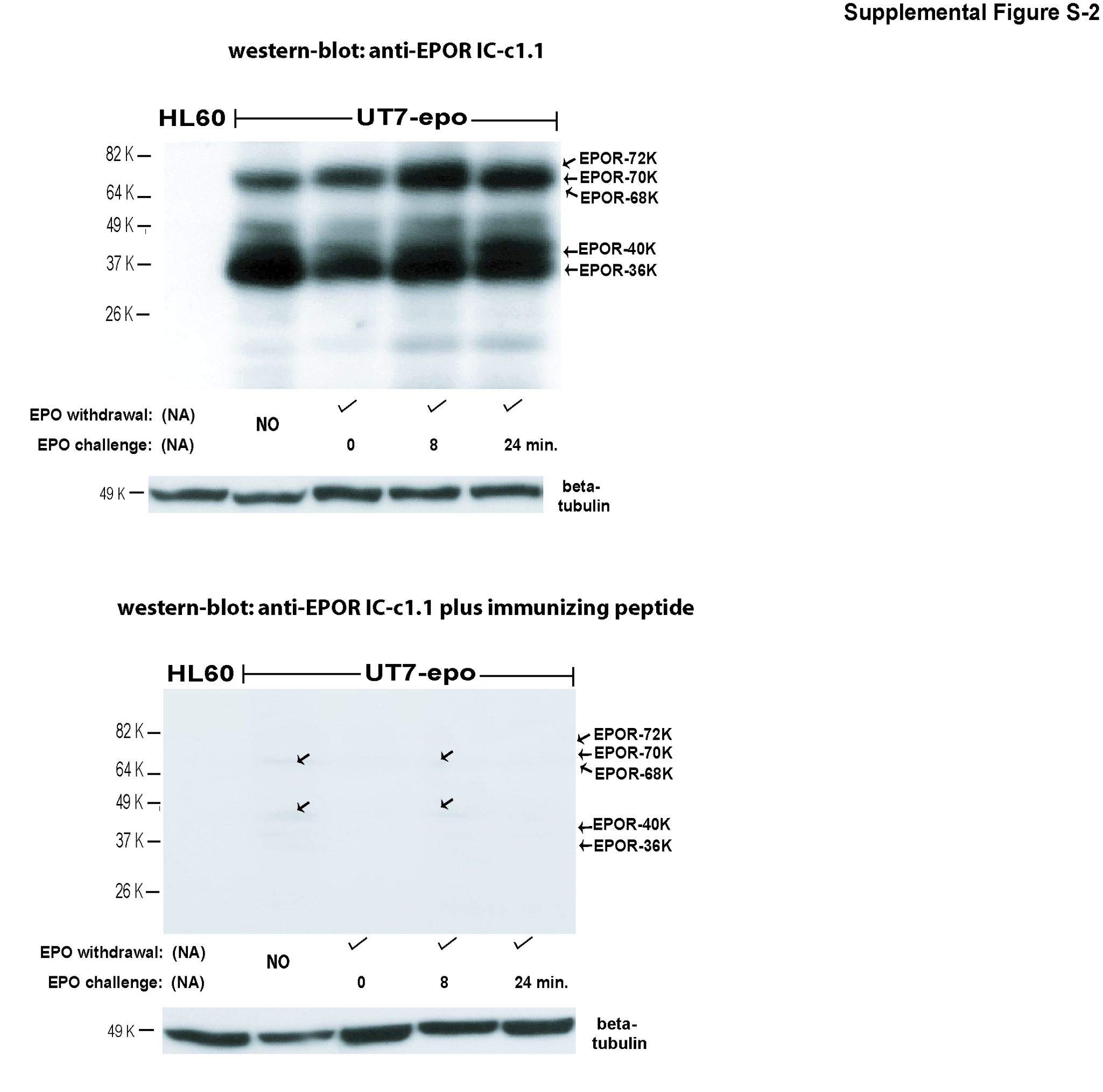

Supplement: Figure S2 — Detection of EPOR molecular species by anti-EPOR antibody IC-c1.1 are uniformly blocked by co-incubation with immunizing peptide. A single gel and western blot was loaded in duplicate (left and right aspects) with the indicated HL60 cell and UT7epo cell lysates. For the half-blot in the lower panel, primary antibody IC-c1.1 was incubated with immunizing peptide (1 µM) during exposure to blotted proteins. Blots were co-processed in parallel. Loading controls are beta-tubulin (for each half blot). (TIF) [file pone.0029064.s002.tif]

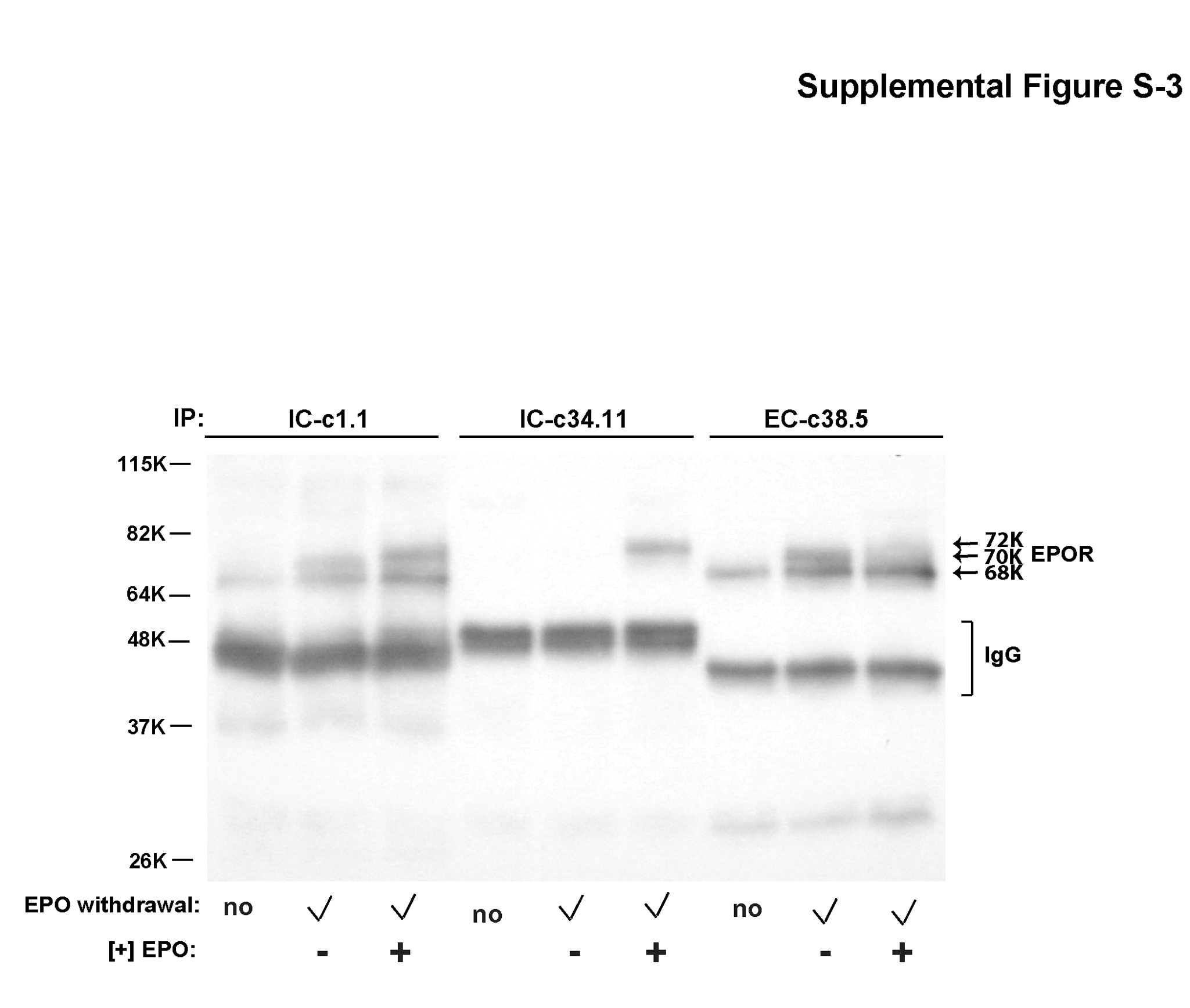

Supplement: Figure S3 — Specific immunoprecipitation of the endogenous hEPOR from UT7epo cells by anti-EPOR antibodies IC-c1.1, IC-c34.11 and EC-c38.5. UT7epo cells were deprived of hematopoietic cytokines for 20 hours. Lysates were then prepared (Igepal, 0.4%) cleared, and incubated for 4 hours with 4 µg of anti-EPOR antibodies (or rabbit IgG). Immune complexes then were retrieved (Protein-A Sepharose CL4B), washed, eluted and analyzed by western blotting with anti-EPOR antibody IC-c1.1. (TIF) [file pone.0029064.s003.tif]

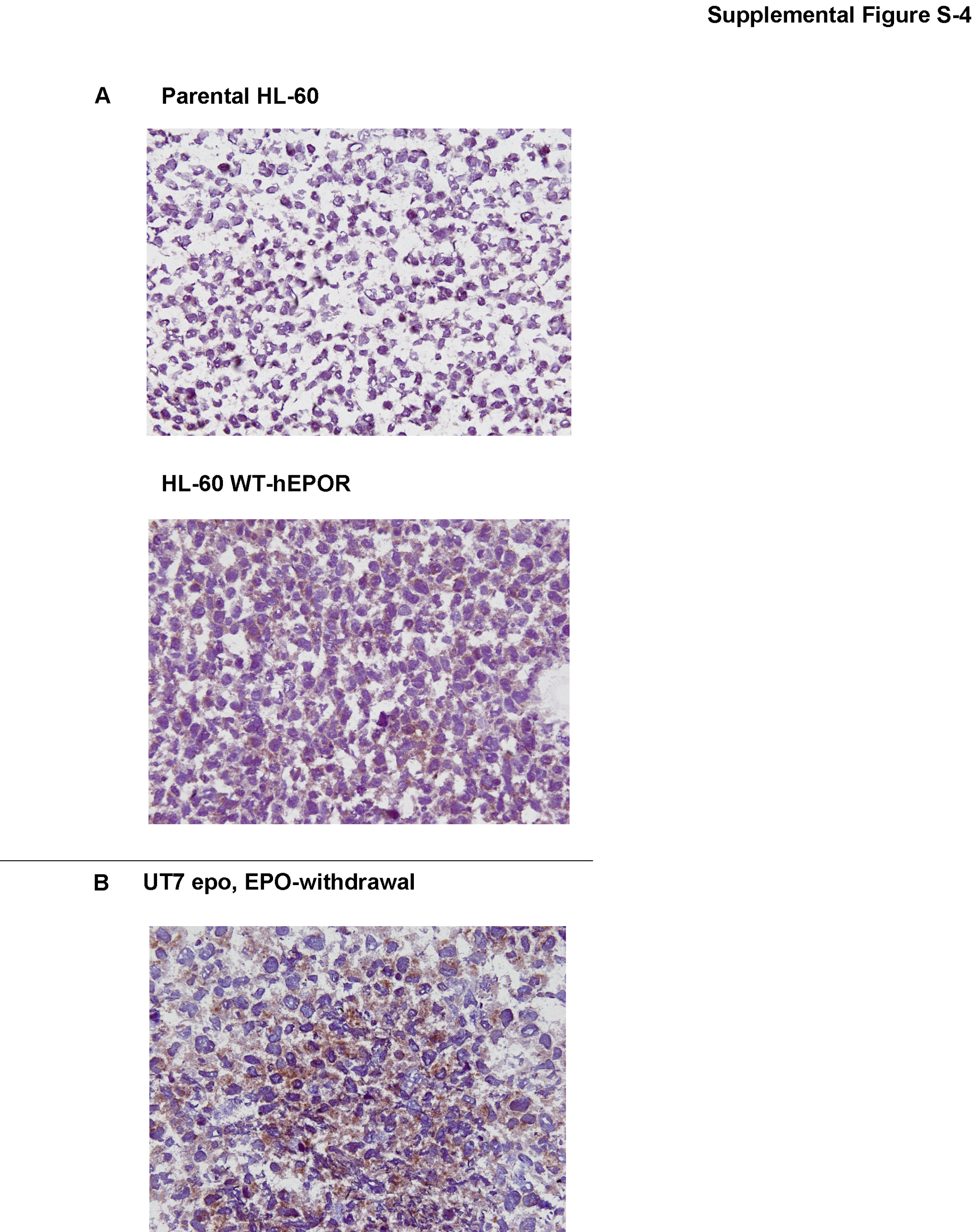

Supplement: Figure S4 — Specific immunohistochemical assays of the human EPOR. A] To allow for optimization of IHC staining (and related IHC preparations) the wild-type EPOR was ectopically expressed at limited levels in EPOR negative myeloid HL60 cells (i.e., levels estimated by western blotting to be approximately 5-fold below those of the endogenous EPOR in UT7epo cells). IHC sections then were prepared as detailed in Methods, and sections were stained with an HRP- coupled anti- rabbit IgG second antibody. B] Using anti-EPOR antibody IC-c1.1, specific IHC assay of the endogenous EPOR in UT7epo cells also was accomplished. (TIF) [file pone.0029064.s004.tif]

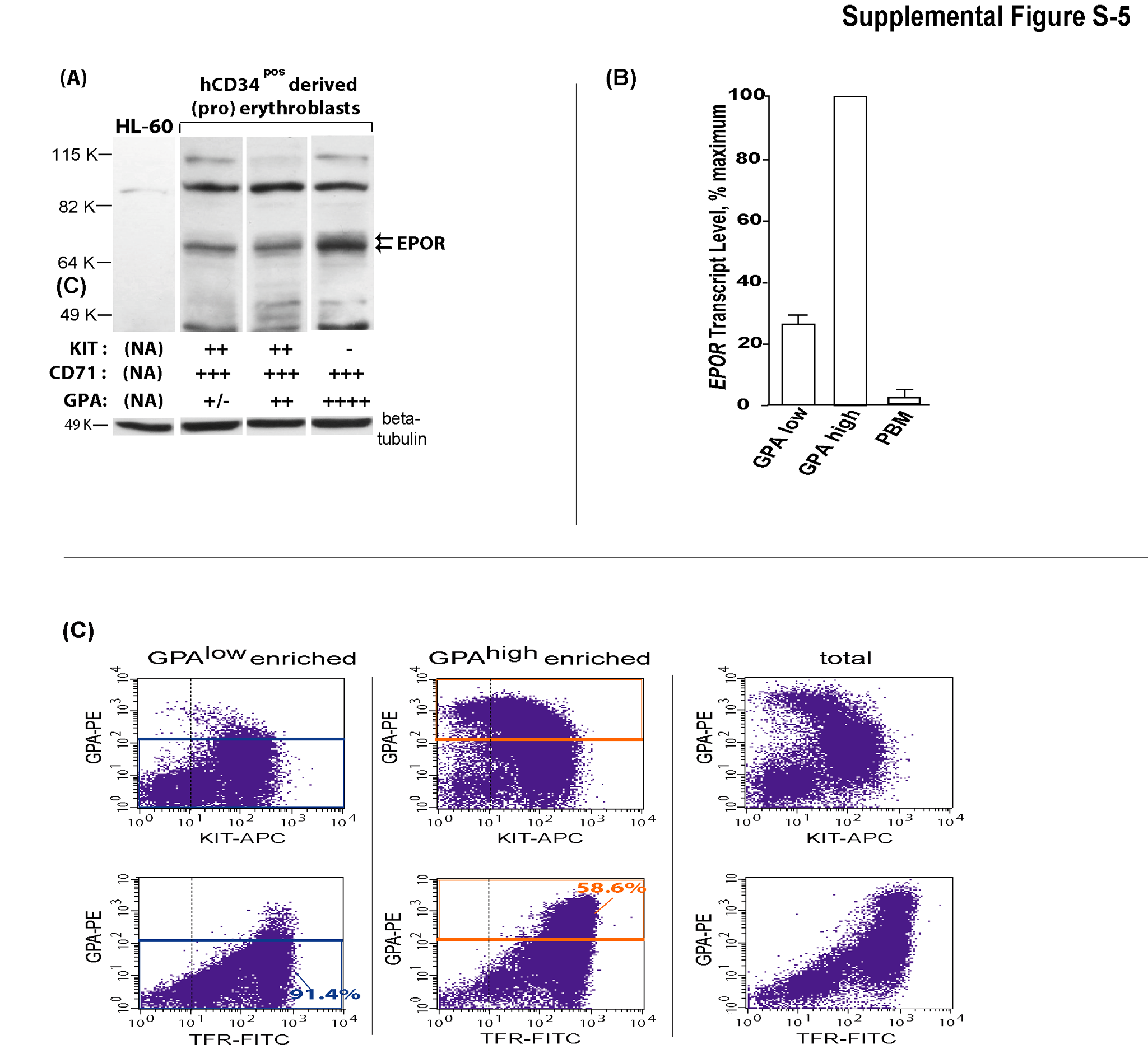

Supplement: Figure S5 — Western blot (and parallel RT-PCR) assays of the EPOR expression in human bone marrow CD34pos progenitor- derived (pro)erythroblasts. Erythroid cells were expanded from human bone marrow CD34pos progenitors in SP34ex medium. At day 10 of expansion, TFR1high (TFR, transferrin receptor) cells were highly represented, and (via MACS) were enriched for GPAlow and GPAhigh sub-populations (lower panel C). EPOR expression levels in each erythroid progenitor pool then were assayed by western blotting (panel A) and quantitative RT-PCR (panel B). (TIF) [file pone.0029064.s005.tif]

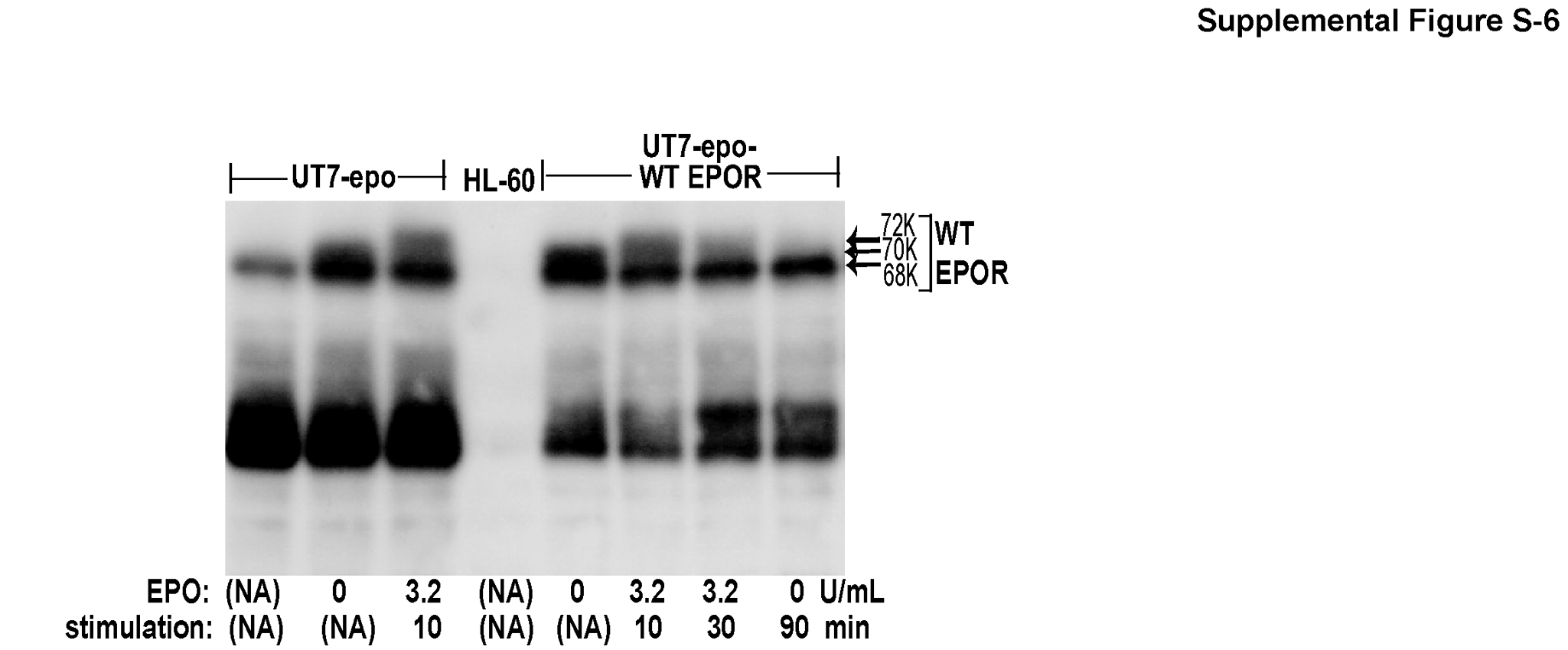

Supplement: Figure S6 — Ectopic expression of EPOR's in UT7epo cells at levels approximating endogenous EPOR levels. UT7epo cells were transduced with VSVG-packaged pMSCVneo viruses encoding hEPOR constructs or no cDNA inset (empty vector). MOI's were varied in order to determine transduction conditions that provided for the expression of EPOR alleles at physiological levels. Western blotting was with anti-EPOR antibody IC-c1.1. Data shown are for parental UT7epo cells vs. stably transduced UT7epo-wtEPOR cells. (Also see Figures 6 and 7). (TIF) [file pone.0029064.s006.tif]

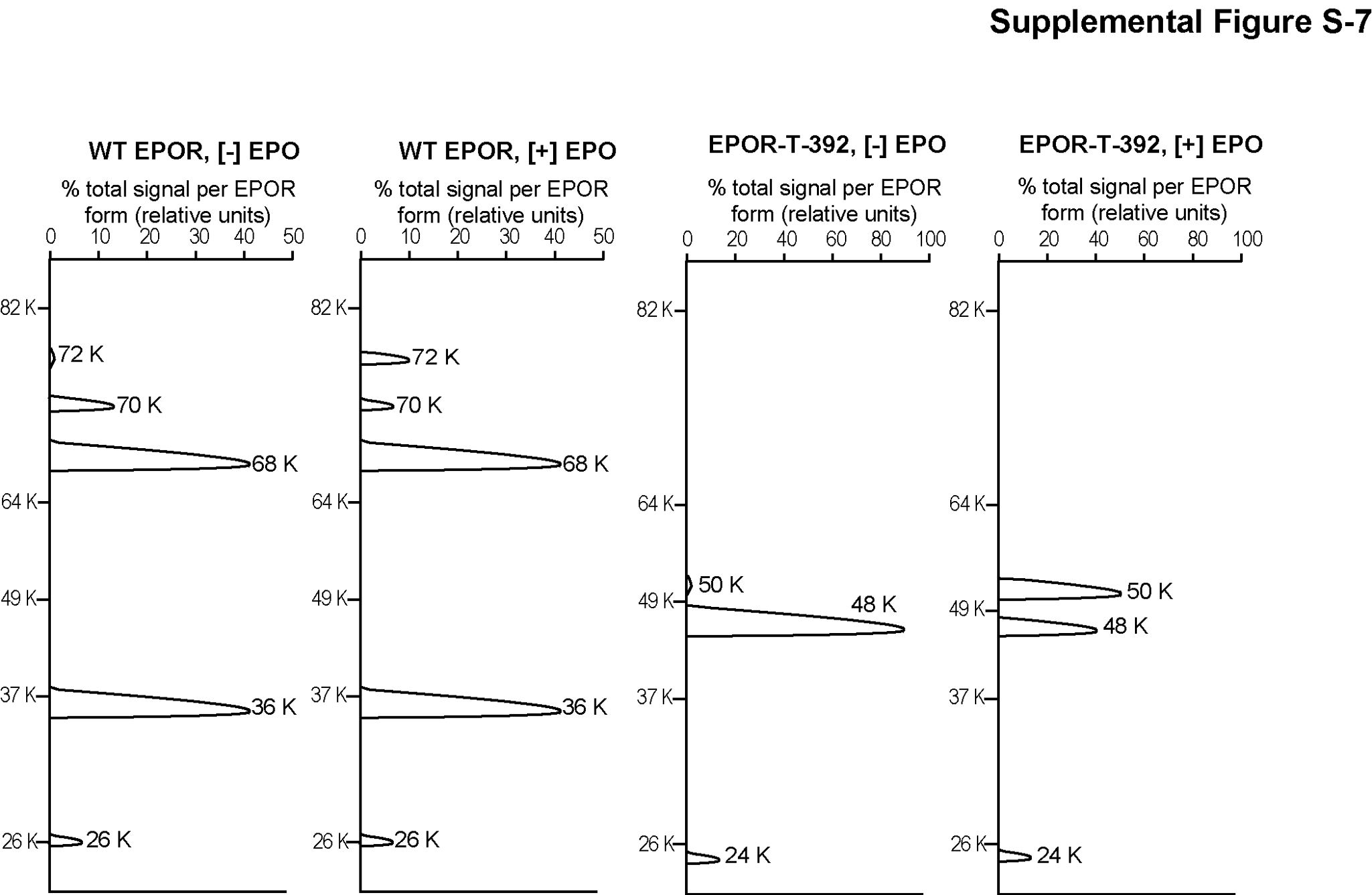

Supplement: Figure S7 — Relative mobilities (in SDS PAGE) of specific EPOR molecular weight species observed upon the expression of the wild-type EPOR and EPOR-T-392 in UT7epo cells. For corresponding western blots, please see Figure 6B. (TIF) [file pone.0029064.s007.tif]

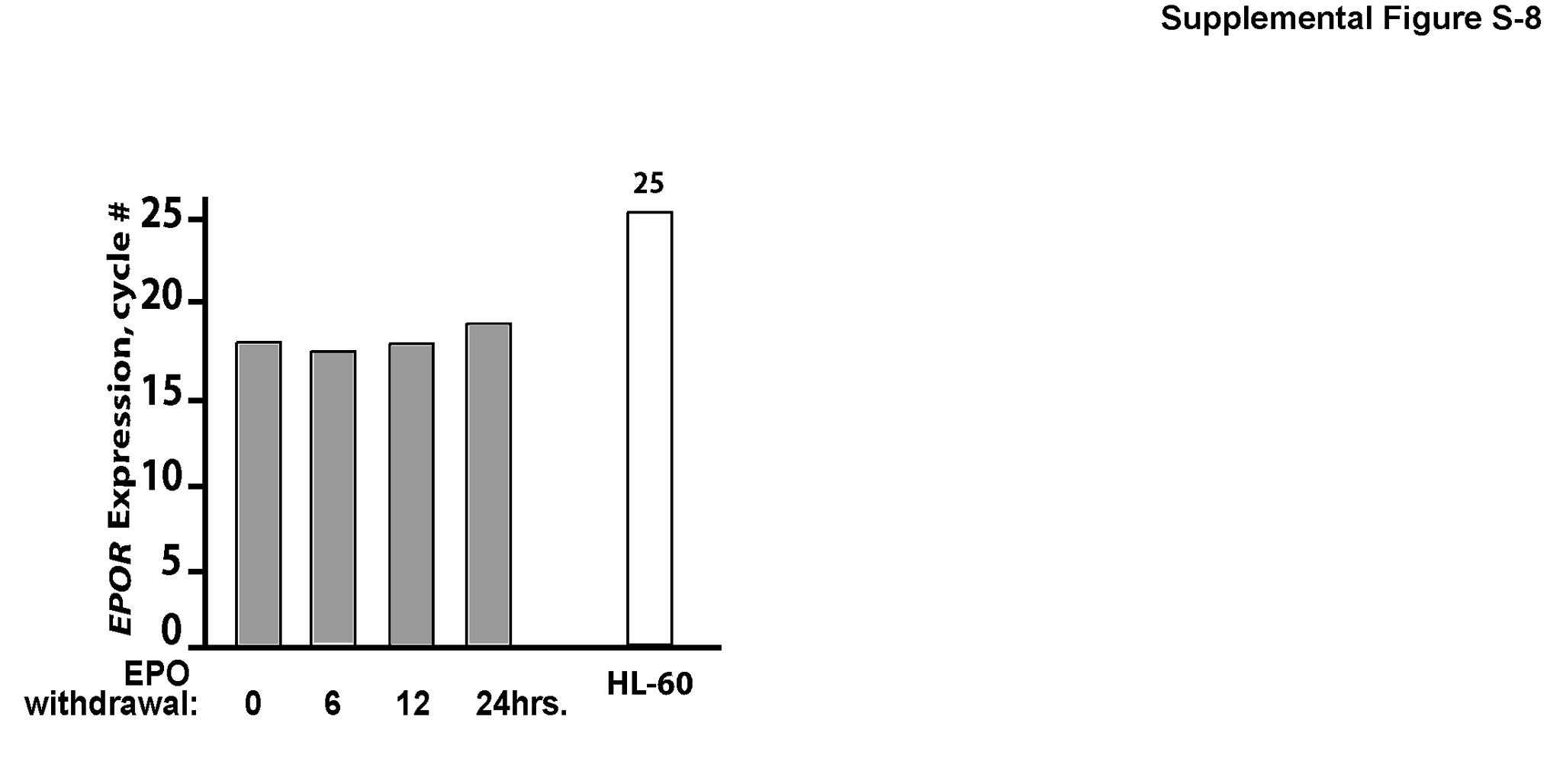

Supplement: Figure S8 — EPO withdrawal does not lead to increases in EPOR transcript levels. Exponentially growing UT7epo cells were plated at 8×10^5^ cells/mL in the absence of hematopoietic cytokines. At 0, 6, 12 and 24 hours of culture, RNA was directly isolated, EPOR transcript levels then were determined by quantitative RT-PCR. Graphed values are cycle numbers normalized for beta-actin (which varied less than 2 cycles overall among samples). (TIF) [file pone.0029064.s008.tif]

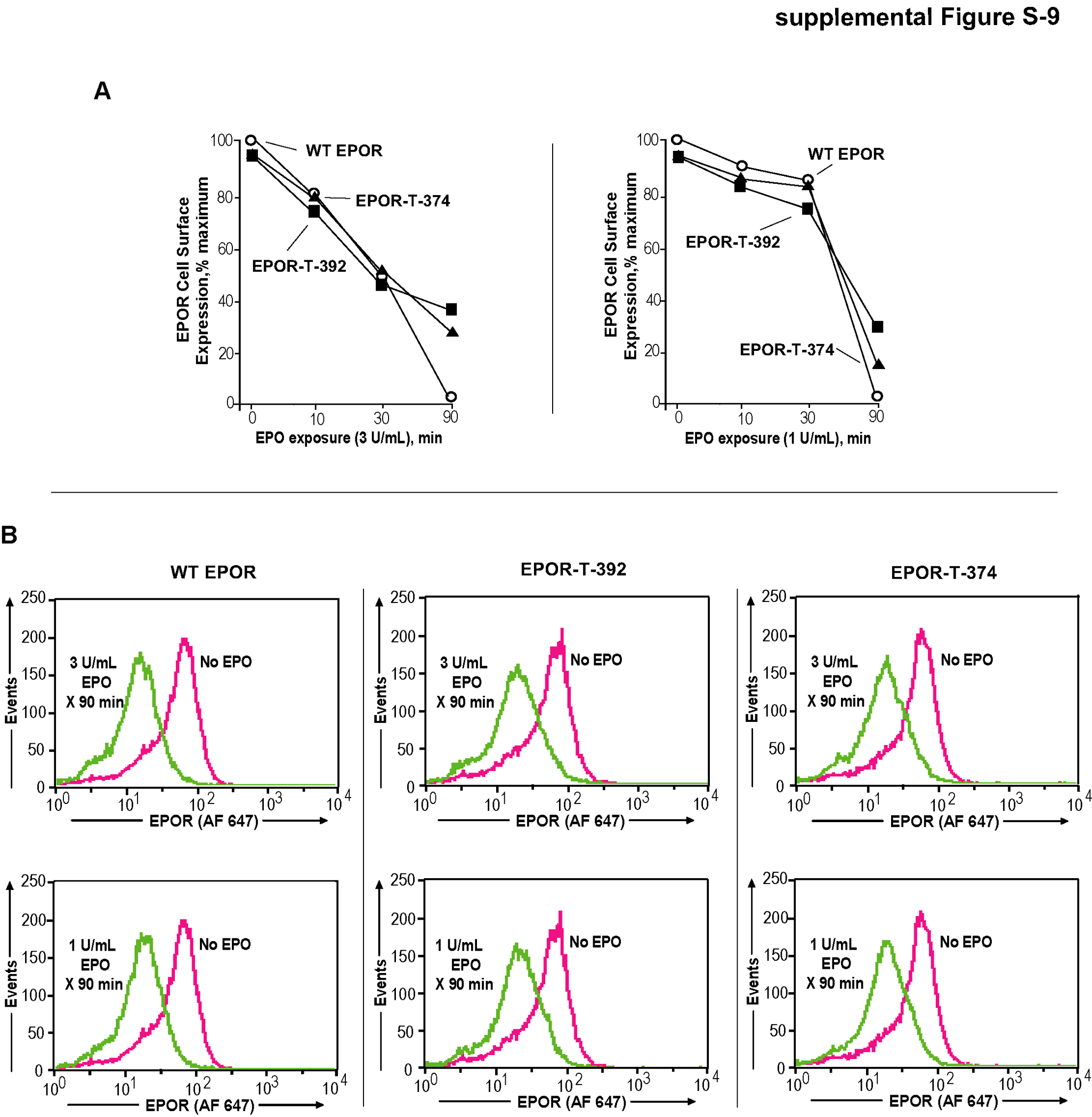

Supplement: Figure S9 — Truncated EPOR alleles harbored by polycythemia patients are moderately attenuated in ligand-induced internalization. A] Attenuated internalization of EPOR-T-392 and EPOR-T-374 alleles – UT7epo cells were transduced (at limiting MOI's) with a pMSCVneo vector encoding the wt-EPOR, EPOR-T-392 or EPOR-T-374. For cells expressing each EPOR form at low, matched levels, ligand-induced rates of EPOR internalization were determined by flow cytometry. This involved EPO withdrawal (20 hours) plus subsequent EPO challenge at either 1 U/mL or 3 U/mL. At 0, 10, 30 and 90 minutes of EPO exposure, levels of cell surface EPOR's were assayed. Truncated EPOR forms underwent internalization, but with apparently attenuated kinetics. B] Primary flow cytometry data are also illustrated (90 minute time points). (TIF) [file pone.0029064.s009.tif]
